# A guide to the BRAIN Initiative Cell Census Network data ecosystem

**DOI:** 10.1371/journal.pbio.3002133

**Published:** 2023-06-30

**Authors:** Michael Hawrylycz, Maryann E. Martone, Giorgio A. Ascoli, Jan G. Bjaalie, Hong-Wei Dong, Satrajit S. Ghosh, Jesse Gillis, Ronna Hertzano, David R. Haynor, Patrick R. Hof, Yongsoo Kim, Ed Lein, Yufeng Liu, Jeremy A. Miller, Partha P. Mitra, Eran Mukamel, Lydia Ng, David Osumi-Sutherland, Hanchuan Peng, Patrick L. Ray, Raymond Sanchez, Aviv Regev, Alex Ropelewski, Richard H. Scheuermann, Shawn Zheng Kai Tan, Carol L. Thompson, Timothy Tickle, Hagen Tilgner, Merina Varghese, Brock Wester, Owen White, Hongkui Zeng, Brian Aevermann, David Allemang, Seth Ament, Thomas L. Athey, Cody Baker, Katherine S. Baker, Pamela M. Baker, Anita Bandrowski, Samik Banerjee, Prajal Bishwakarma, Ambrose Carr, Min Chen, Roni Choudhury, Jonah Cool, Heather Creasy, Florence D’Orazi, Kylee Degatano, Benjamin Dichter, Song-Lin Ding, Tim Dolbeare, Joseph R. Ecker, Rongxin Fang, Jean-Christophe Fillion-Robin, Timothy P. Fliss, James Gee, Tom Gillespie, Nathan Gouwens, Guo-Qiang Zhang, Yaroslav O. Halchenko, Nomi L. Harris, Brian R. Herb, Houri Hintiryan, Gregory Hood, Sam Horvath, Bingxing Huo, Dorota Jarecka, Shengdian Jiang, Farzaneh Khajouei, Elizabeth A. Kiernan, Huseyin Kir, Lauren Kruse, Changkyu Lee, Boudewijn Lelieveldt, Yang Li, Hanqing Liu, Lijuan Liu, Anup Markuhar, James Mathews, Kaylee L. Mathews, Chris Mezias, Michael I. Miller, Tyler Mollenkopf, Shoaib Mufti, Christopher J. Mungall, Joshua Orvis, Maja A. Puchades, Lei Qu, Joseph P. Receveur, Bing Ren, Nathan Sjoquist, Brian Staats, Daniel Tward, Cindy T. J. van Velthoven, Quanxin Wang, Fangming Xie, Hua Xu, Zizhen Yao, Zhixi Yun, Yun Renee Zhang, W. Jim Zheng, Brian Zingg

**Affiliations:** 1 Allen Institute for Brain Science, Seattle, Washington, United States of America; 2 Department of Neuroscience, University of California San Diego, San Diego, California, United States of America; 3 San Francisco Veterans Affairs Medical Center, San Francisco, California, United States of America; 4 Bioengineering Department and Center for Neural Informatics, Structures, & Plasticity, Volgenau School of Engineering, George Mason University, Fairfax, Virginia, United States of America; 5 Institute of Basic Medical Sciences, University of Oslo, Oslo, Norway; 6 UCLA Brain Research & Artificial Intelligence Nexus, Department of Neurobiology, David Geffen School of Medicine at University of California, Los Angeles, California, United States of America; 7 McGovern Institute for Brain Research, Massachusetts Institute of Technology, Cambridge, Massachusetts, United States of America; 8 Department of Physiology, University of Toronto, Toronto, Ontario, Canada; 9 Department of Otorhinolaryngology Head and Neck Surgery, University of Maryland School of Medicine, Baltimore, Maryland, United States of America; 10 Department of Anatomy and Neurobiology, University of Maryland School of Medicine, Baltimore, Maryland, United States of America; 11 Institute for Genome Sciences, University of Maryland School of Medicine, Baltimore, Maryland, United States of America; 12 Department of Radiology, University of Washington, Seattle, Washington, United States of America; 13 Nash Family Department of Neuroscience and Friedman Brain Institute, Icahn School of Medicine at Mount Sinai, New York, New York, United States of America; 14 Department of Neural and Behavioral Sciences, College of Medicine, The Pennsylvania State University, Hershey, Pennsylvania, United States of America; 15 SEU-Allen Institute Joint Center, Institute for Brain and Intelligence, Southeast University, Nanjing, Jiangsu Province, China; 16 Cold Spring Harbor Laboratory, Cold Spring Harbor, New York, United States of America; 17 Department of Cognitive Science, University of California, San Diego, La Jolla, California, United States of America; 18 European Bioinformatics Institute (EMBL-EBI), Wellcome Trust Genome Campus, Hinxton, Cambridge, United Kingdom; 19 Genentech, South San Francisco, California, United States of America; 20 Pittsburgh Supercomputing Center, Carnegie Mellon University, Pittsburgh, Pennsylvania, United States of America; 21 J. Craig Venter Institute, La Jolla, California, United States of America; 22 Data Sciences Platform, Broad Institute of MIT and Harvard, Cambridge, Massachusetts, United States of America; 23 Feil Family Brain and Mind Research Institute, Weill Cornell Medicine, New York, New York, United States of America; 24 Research and Exploratory Development Department, Johns Hopkins University Applied Physics Laboratory, Laurel, Maryland, United States of America; 25 Chan Zuckerberg Initiative, Redwood City, California, United States of America; 26 Kitware Inc., Albany, New York, United States of America; 27 Department of Biomedical Engineering, Johns Hopkins University, Baltimore, Maryland, United States of America; 28 CatalystNeuro, Benicia, California, United States of America; 29 Department of Radiology, Perelman School of Medicine, University of Pennsylvania, Philadelphia, Pennsylvania, United States of America; 30 Genomic Analysis Laboratory, Howard Hughes Medical Institute, The Salk Institute for Biological Studies La Jolla, California, United States of America; 31 Bioinformatics and Systems Biology Graduate Program, University of California San Diego, La Jolla, California, United States of America; 32 Texas Institute for Restorative Neurotechnologies, The University of Texas Health Science Center at Houston, Houston, Texas, United States of America; 33 Department of Psychological and Brain Sciences, Dartmouth College, Hannover, New Hampshire, United States of America; 34 Environmental Genomics and Systems Biology Division, Lawrence Berkeley National Laboratory, Berkeley, California, United States of America; 35 Department of Intelligent Systems, Delft University of Technology, Delft, the Netherlands; 36 Department of Radiology, Leiden University Medical Center, Leiden, the Netherlands; 37 Center for Epigenomics, Department of Cellular and Molecular Medicine, UC San Diego School of Medicine, La Jolla, California, United States of America; 38 Ludwig Institute for Cancer Research, La Jolla, California, United States of America; 39 Microsoft Corporation, Seattle, Washington, United States of America; 40 UCLA Brain Mapping Center, University of California, Los Angeles, California, United States of America; 41 Department of Chemistry and Biochemistry, University of California Los Angeles, California, United States of America; 42 School of Biomedical Informatics, The University of Texas Health Science Center at Houston, Houston, Texas, United States of America

## Abstract

Characterizing cellular diversity at different levels of biological organization and across data modalities is a prerequisite to understanding the function of cell types in the brain. Classification of neurons is also essential to manipulate cell types in controlled ways and to understand their variation and vulnerability in brain disorders. The BRAIN Initiative Cell Census Network (BICCN) is an integrated network of data-generating centers, data archives, and data standards developers, with the goal of systematic multimodal brain cell type profiling and characterization. Emphasis of the BICCN is on the whole mouse brain with demonstration of prototype feasibility for human and nonhuman primate (NHP) brains. Here, we provide a guide to the cellular and spatial approaches employed by the BICCN, and to accessing and using these data and extensive resources, including the BRAIN Cell Data Center (BCDC), which serves to manage and integrate data across the ecosystem. We illustrate the power of the BICCN data ecosystem through vignettes highlighting several BICCN analysis and visualization tools. Finally, we present emerging standards that have been developed or adopted toward Findable, Accessible, Interoperable, and Reusable (FAIR) neuroscience. The combined BICCN ecosystem provides a comprehensive resource for the exploration and analysis of cell types in the brain.

## Abstract 2

Characterizing cellular diversity is necessary to understand the function of different cell types in the brain. This Consensus View provides a guide to the cellular and spatial approaches used in cell type surveys by the BRAIN Initiative Cell Census Network (BICCN), as well as information on accessing and using the BICCN data and its extensive resources.

## Introduction and overview

The National Institutes of Health’s Brain Research Through Advancing Innovative Neurotechnologies (BRAIN) Initiative, launched in 2013, is a major effort to accelerate neuroscience research by providing researchers with tools to study and treat human brain disorders through a comprehensive understanding of the human brain [1]. Following a pilot phase [2] surveying the feasibility of scaling single-cell profiling technologies, the BRAIN Initiative Cell Census Network (BICCN) launched a 5-year phase (2017 to 2022), with the goal of systematic multimodal cell type profiling and characterization of the whole mouse brain, with parallel proof of concept for a similar characterization and scalability to tackle the much larger human and nonhuman primate (NHP) brains. This effort resulted in broad collaboration among the neuroscience community to apply advanced single-cell profiling to characterize transcriptomic and epigenomic signatures, anatomical phenotypes, and functional properties of brain cell types and accelerated the rapid sharing of cell census data with the larger community prepublication. The success of these efforts is built on significant advances in scalable single-cell analysis including single-cell genomic (RNA, ATAC-seq, and methylation) profiling, anatomical mapping at cellular resolution, and other approaches and has proven to be powerful and scalable. The BICCN has completed whole mouse brain RNA-seq and spatial transcriptomic atlases [3,4], and large-scale research in human and NHP atlases has begun through the newly initiated BRAIN Initiative Cell Atlas Network (BICAN) [5]. These resulting data resources are already proving invaluable for researchers across many areas of neuroscience. Here, we provide a comprehensive description and user guide to available resources and discuss how they can enable rapid progress in neuroscience.

BICCN is a collaborative network of centers and laboratories, including data generating centers, data archives, and data standards developers, which generate, map, and share resources to support several overarching goals. These include generating a high-resolution, comprehensive atlas of cell types in the mouse brain based on large-scale single-cell transcriptome and epigenome sequencing, along with systematic characterization of neuronal morphology, a census of the number and location of cells for each type, new genetic tools to experimentally target brain cell types, and a prototype atlas of human brain and NHP cell types in selected regions of the adult and developing human brain. A standard anatomical template for mapping cell types in the mouse brain was also established through completion and validation of a common coordinate framework (CCF; [6]). BICCN also conducted an initial profiling of cellular diversity in several structures relevant to neurodegenerative and neuropsychiatric disease, including the hippocampus and dorsolateral prefrontal cortex, and, importantly, cross-species identification and mapping of cell types between mouse, marmoset, and human (S1 Text—BICCN Scientific Outcomes).

Each BICCN project has contributed publicly accessible data to multimodal classification of cell types based on transcriptomic, epigenetic, proteomic, morphological connectivity, anatomic distribution, and physiological signatures of cells for further study. To date, the BRAIN Initiative data archives store petabytes of omics, imaging, and neurophysiology datasets generated using over 40 cell profiling techniques and 97 published protocols (see Data Archives for the BICCN, BICCN Data Processing Pipelines). The BICCN BRAIN Cell Data Center (BCDC; biccn.org) manages this ecosystem, together with data archives to support logistical organization, data integration, and development of common data standards as well as central maintenance to sustain, compare, and reanalyze data. A major success of the BICCN has been to embrace an operating principle that data should be released quarterly, prepublication, and freely shared under CC-BY-4.0 license unless human protection restrictions apply. In this way, the BICCN Data Ecosystem represents one of the largest resources for single-cell data of the brain and any organ.

The first phase of the BICCN generated a comprehensive multimodal cell census and atlas of the mammalian primary motor cortex (MOp or M1) [7]. This project involved coordinated large-scale analyses of single-cell transcriptomes [8,9], chromatin accessibility [10], DNA methylomes [11], spatially resolved single-cell transcriptomes [12], anatomic characterization with morphological and electrophysiological properties [13,14], and cellular resolution input–output mapping [10,15]. The results and their extension to the whole mouse brain and other human regions represent a milestone in the effort to create a catalog or census of all brain cell types and advance the collective knowledge and understanding of brain cell type organization. Six active BICCN Working Groups continue to extend and integrate new and existing data across labs toward an integrated transcriptomic and epigenomic atlas of the entire mouse central nervous system.

BICCN reflects the increasingly collaborative nature of modern neuroscience and has accomplished the deepest coordinated characterization of cell types in any organ to date. Consortia such as the Human Cell Atlas (HCA; [16]) and Human Biomolecular Atlas Program [17] are also key representatives of this community and are leading molecular profiling in other organs. Here, we describe the BICCN data ecosystem and provide a guide to accessing and using its data and resources. The section Characterizing Cell Types of the Brain describes the challenge of brain cell type profiling, the approaches taken by BICCN investigators, and requirements for spatial localization and data architecture. The BICCN Data Ecosystem section overviews the data ecosystem and the BCDC and its role in data management. Data Archives for the BICCN provides a guide to the primary BICCN-related data archives describing methods for accessing archived data and the process of data submission. In BICCN Data Processing Pipelines, we describe progress in standardizing molecular and image-based processing pipelines and their use. We offer several usage vignettes and describe some of the many BICCN tools for analysis and visualization that have been developed in the section Working with BICCN Data. Standards that have been developed or adopted within the BICCN are described in Standards and the BICCN: Towards FAIR Neuroscience, which provides an inventory of progress in Findable, Accessible, Interoperable, and Reusable (FAIR) [18] neuroscience. Finally, S1–S4 Tables provide information throughout the guide on the many resources available to users of these rich data.

### Characterizing cell types of the brain

Understanding reproducible features of brain cells is a prerequisite to characterize cell types and to understand their function in the brain, to manipulate them in controlled ways, and to determine variability in brain disorders. Neurons can be distinguished by differential expression of gene classes such as neurotransmitters and neuropeptides, electrophysiological firing patterns, morphology, and by their connectivity, and these modalities form a natural basis for classification. Properties of glial cells, vascular cells, and immune cells in the nervous system are also essential to understand brain function in health and disease [19]. Moreover, the brain has an immensely complex global and regional structure and mapping the distribution of cell types across regions, and nuclei is a vital part of characterization.

### BICCN cell type profiling

There is general agreement that types should be defined by invariant and generally intrinsic properties and that this classification can provide a good starting point for a census [7]. There are, however, significant challenges in characterizing cell types because of inherent biological variability, imperfect measurements, and challenges of data integration between modalities [7,20]. While past attempts have not resulted in a unified taxonomy of neuronal or glial cell types, partly due to limited data, single-cell transcriptomics is enabling, for the first time, systematic high-throughput measurements of brain cells and generation of datasets that hold the promise of being complete, accurate, and permanent [21]. However, the structure and relationships of cell types is very complex with evidence that there are not always sharp boundaries separating different regions, particularly in the cerebral cortex [22]. For a recent overview of brain cell type profiling and its challenges, see [23].

**Fig 1:**
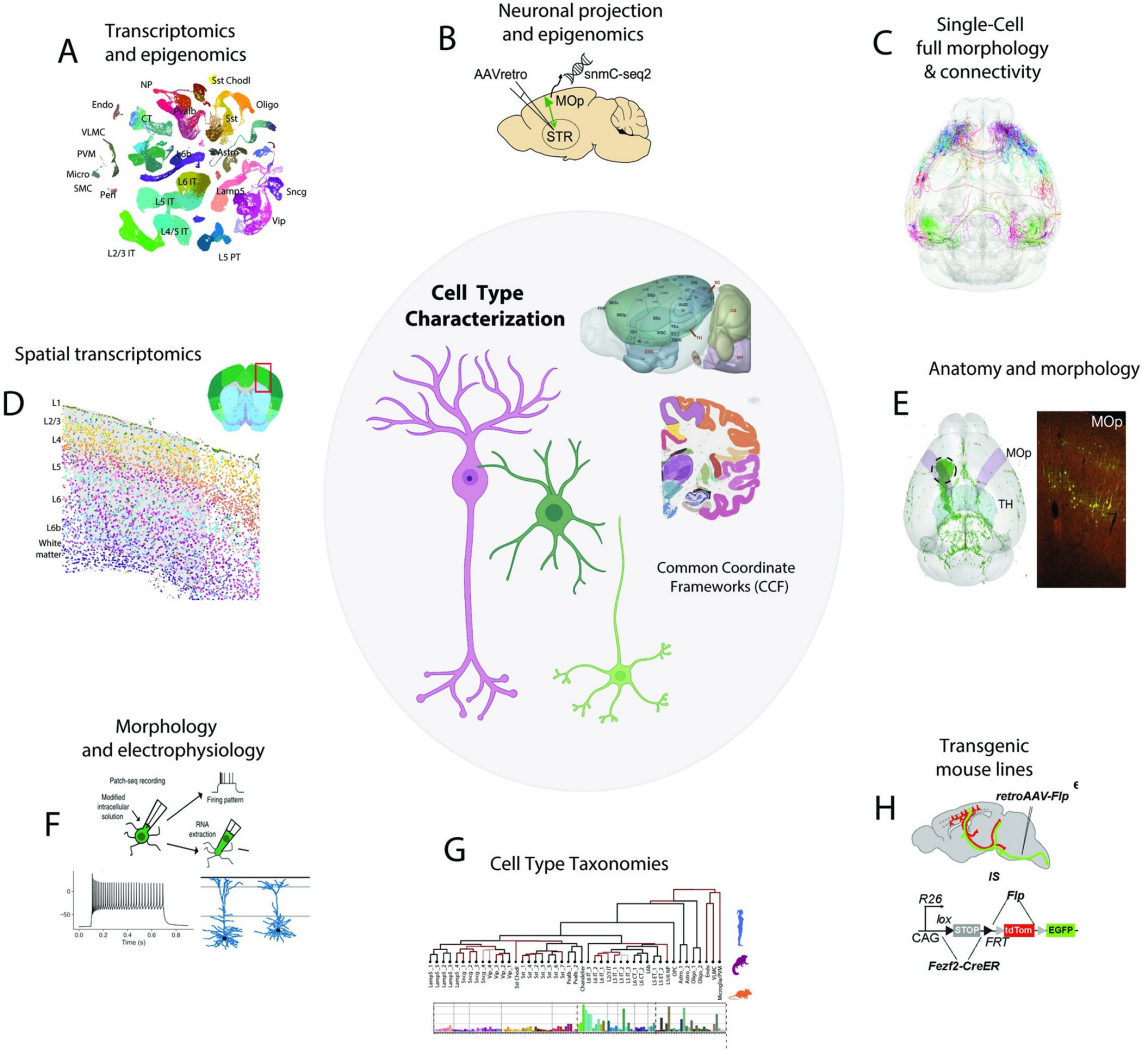
Cell type profiling and major approaches. A variety of multimodal techniques are used to profile cell types of the brain. A common coordinate framework (CCF) is used to map spatial distribution of types and their connectivity. Top to bottom: (A) Transcriptomic techniques, single-cell and single-nucleus (sc/sn-RNA-seq), and epigenomic (ATAC-seq), single-nucleus methylation (snmC-seq), (B) epi-retro-seq, (C) single-cell full morphology and connectivity (fMOST, BAR-seq), (D) spatial transcriptomics (MERFISH), (E) antero- and retro-grade tracing methods for morphological reconstruction. (F) Multimodal technique combining transcriptome, electrophysiology, and morphology (Patch-seq). (G) Cell type classifications are represented as taxonomies reflecting hierarchical relationships, multimodal correspondence, and cell distribution (S1 Table). (H) Transgenic mouse lines are used in selecting expressing cell types.

A full characterization of cell types for a given brain region will consist of an enumeration of distinct types characterized across different biological features, including the distributions of their molecular profiles (transcriptome, proteome, chromatin accessibility), developmental history, morphology, functionality (e.g., electrophysiology), and spatial location mapped to a CCF or atlas of the brain. The result of profiling in this way is the development of a taxonomy of different types derived from multiple modality data and their congruences (Fig 1). Present classification studies approach but generally do not fully attain complete characterization, due to having only partial measurements and lacking full data correspondences. Determining relative significance of data is challenging, and while each modality is valuable, the transcriptome forms a natural template to which other modalities can be mapped for completion and essential missing information.

**Fig 2:**
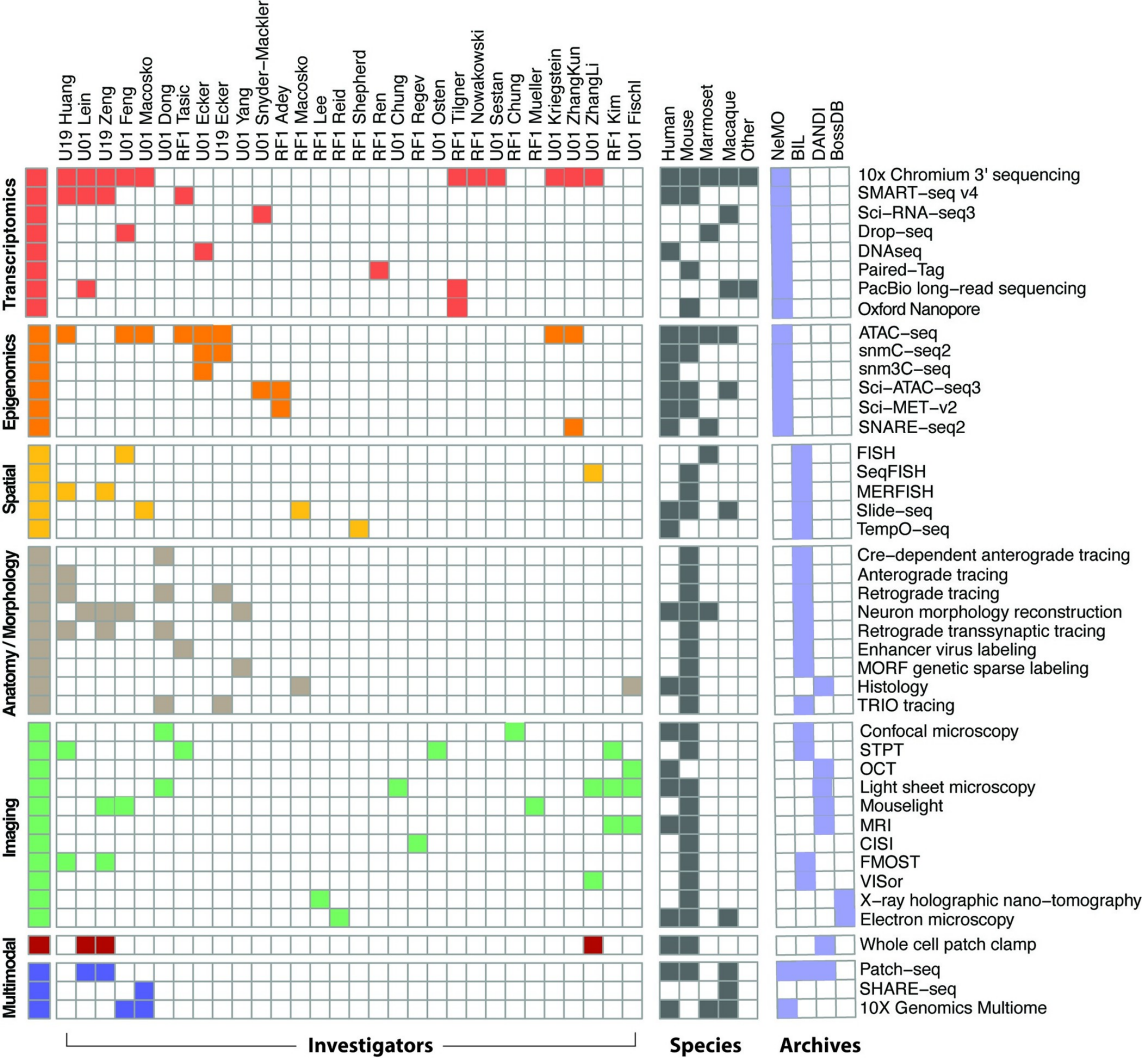
BICCN cell type modalities, techniques, and investigators. Primary techniques (right annotation) used in profiling cell types by BICCN investigators (top) are colored by major modality (left) and primary species (S1 Table). Investigator awards are ordered by techniques common to laboratories. BRAIN Initiative data archives store primary data shown by modality; NeMO, Neuroscience Multi-Omic Archive; BIL, Brain Imaging Library; DANDI, Distributed Archives for Neurophysiology Data Integration; BossDB, Brain Observatory Storage Service and Database (see Data archives for the BICCN); The NIH UM1, cooperative agreements involving large-scale research activities; U19, multidisciplinary with specific major objective; U01, discrete, specified, circumscribed project; RF1, discrete, specific project by named investigator (NIH Grants).

To progress toward this goal, BICCN studies used a wide array of approaches (Figs 1 and 2; S1 Table), broadly classified as single-cell transcriptomic and epigenomics, spatial transcriptomics, anatomy/morphology, imaging-based, electrophysiology, and multimodal, spanning more than 40 high-resolution methods for investigation of cell type characteristics. Some of the most broadly used BICCN methods (Fig 1) include single-cell and single-nucleus RNA-seq (sc/snRNA-seq) [8,24–26], single-nucleus long-read sequencing [27], single-cell ATAC-seq [8], snmC-seq [11], epi-retro-seq [15], single-cell full morphology and BAR-seq [28], MERFISH and other spatial transcriptomics methods [12], anterograde and retrograde tracing for morphology [14], multimodality Patch-Seq [12,13], and the use of transgenic lines [29]. All data in the mouse were mapped to the CCF through either image registration or specimen pinning (See Common coordinate frameworks of the brain).

The cell type profiling techniques developed and used by the more than 30 BICCN projects are presented in Fig 2, illustrating the breadth of the consortium’s approaches. These techniques are broadly classified as transcriptomic, epigenomic, spatial transcriptome, anatomy/morphology, imaging-based, electrophysiology, and multimodal, spanning a wide range of more than 40 high-resolution methods for investigation of cell type characteristics. Investigators are grouped here by techniques common to their programs. While the primary focus of the BICCN is on the mouse, Fig 2 shows profiling applied to human, marmoset, and macaque as well as several other species for an evolutionary study [9]. S1 Table provides details on the primary techniques used and BICCN investigator projects (see also Team Pages on biccn.org).

### BICCN data levels

The importance of having structure in data grows with increasing annotation and its association with existing knowledge. The hierarchical organization of information is an active area of bioinformatics [30,31]. Among other benefits, the specification of the structure of a dataset and its relevant metadata provides a mechanism for efficient retrieval of datasets by users. BICCN data and structured datasets are classified through Data Levels (Fig A in S1 Text), reflecting a common conceptual approach for identifying increasing levels of structure from data, through information, to knowledge [32]. In this way, BICCN datasets are classified by information content ranging from primary Raw (Level 0) data directly from individual laboratories running specific assay platforms, to QC/QA Validated (Level 1) data with appropriate associated metadata, Linked (Level 2) data that are associated with a specific brain region or nuclei, datasets with computed Features (Level 3), and, finally, Integrated (Level 4) datasets having biological relevant annotation and comparison with other sources (see S1 Text—BICCN Data Levels).

Data Levels are more than a labeling system and provide an entry point for users of the BICCN data corpus and use–case-directed identification of datasets of particular interest. At project award, each BICCN investigator specified levels of data that their project would generate and BICCN working groups collectively reconcile these definitions by each modality such as 10×-snRNA seq, MERFISH, or electrophysiology to achieve modality specific definitions across groups (S2 Table). While all Level 1 data are required to be deposited in BRAIN Initiative archives (see Data archives for the BICCN) on a quarterly basis, uniform storage and archiving requirements for datasets with more structure are currently being developed by the BRAIN archives as required by BICCN program objectives. S3 Table lists current BICCN-level classified datasets and their provenance. There is some flexibility in defining levels particularly with increasing annotation and structure.

### Common coordinate frameworks of the brain

**Fig 3:**
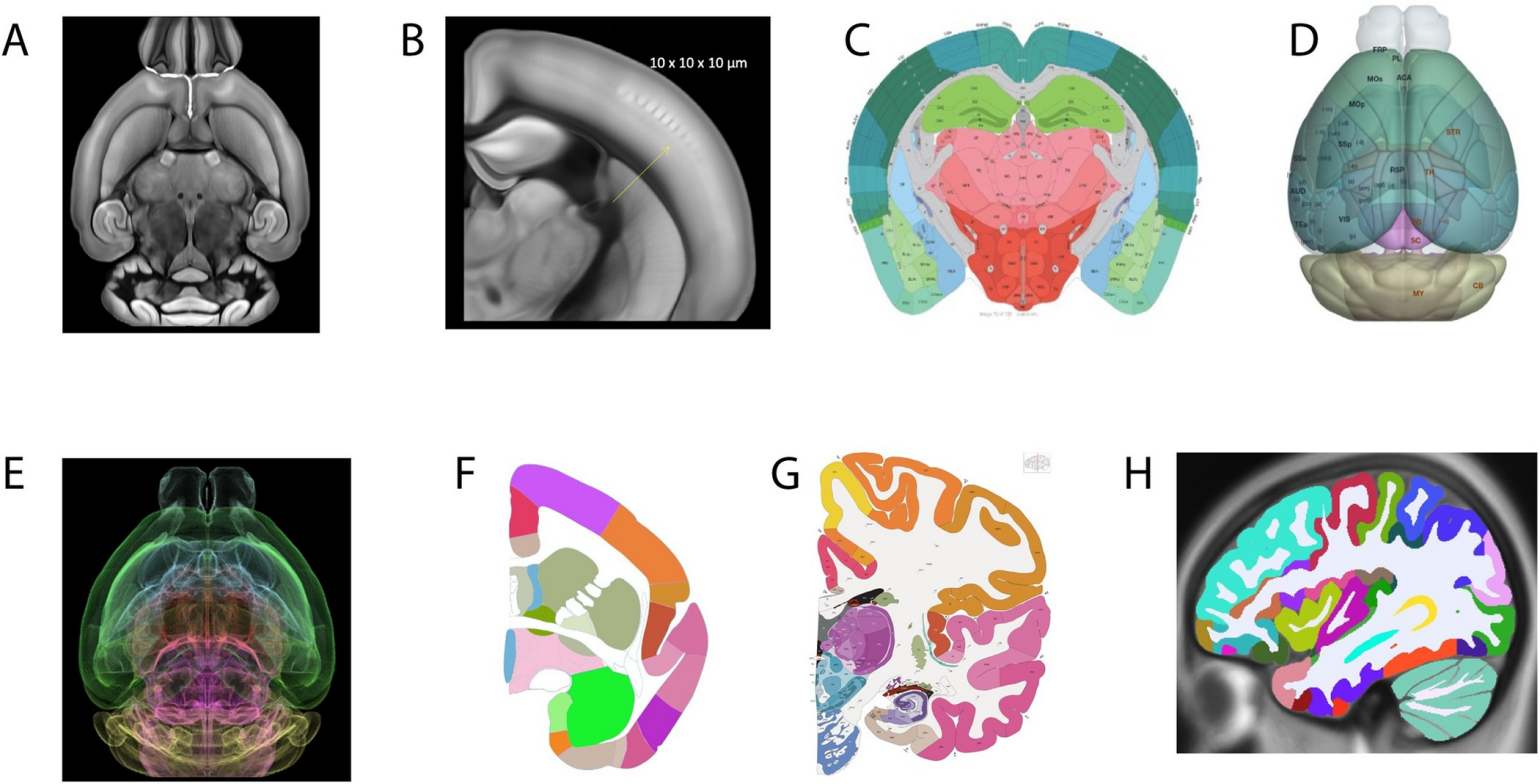
Common coordinate frameworks of the brain. **(**A, B) Allen Mouse Brain Common Coordinate Framework (CCF) constructed from serial 2-photon tomography images with 100 μm z-sampling from 1,675 young adult C57BL/6J mice yields 10-μm cubic resolution. (C) Digital atlases of the Mouse (Allen CCFv3) annotated plate and (D) 3D reconstruction. (E) fMOST mouse atlas derived from CCFv3 through iterative averaging of 36 fMOST brains. This approach to a reference atlas reduces the average distance error of somata mapping up to 40% (F) marmoset atlas plate (Allen Institute for Brain Science). (G) Human reference atlas from 34-year-old female, 1 mm/pixel Nissl and immunohistochemistry anatomical plates, annotated 862 structures, including 117 white matter tracts and several novel cyto- and chemoarchitecturally defined structures. (H) MRI-based annotation of human atlas of 150 structures form the initial atlas for BICAN profiling.

Spatially localizing cell type data to a CCF provides an anatomical context that is essential to understand the role of cell types in brain function. When spatially mapped, data achieve Linked data level and allow users to identify and access data in a regionally annotated way. BICCN data from the mouse brain are mapped to the Allen Mouse Common Coordinate Framework (http://atlas.brain-map.org/; [6]), which serves as the main anatomic data browser and spatial coordinate environment for mouse data, as well as the reference atlas for mouse data within EBRAINS, the European infrastructure for brain and brain-inspired research (https://ebrains.eu/service/mouse-brain-atlas). CCFv3 is based on a 3D 10-μm isotropic, highly detailed population average of 1,675 mouse brains using 2-photon imaging (Fig 3A and 3B) and consists of 207 newly drawn structures in 3D: 123 subcortical structures, 41 fiber tracts (plus ventricular systems), and 43 cortical regions, including primary visual and higher visual areas. Ultimately, more than 500 gray matter structures, cortical layers, approximately 80 fiber tracts, and ventricle structures in 3D will be included [6] (Fig 3C and 3D). A recent fMOST atlas was derived from CCFv3 [33], extending registration accuracy for this modality (Fig 3E). The CCFv3 is being refined and improved through the BICCN and currently provides a definitive mouse brain reference framework.

Human and NHP atlases are similarly necessary for structure identification and data mapping (Fig 3F–3H). However, here, current reference atlases have major limitations such as lack of whole-brain coverage, relatively low image resolution, and sparse structural annotation. The BICCN uses the Allen Human Reference Atlas - 3D, 2020, a human brain atlas [34] that incorporates neuroimaging, high-resolution histology, and chemo architecture across a complete adult female brain, with magnetic resonance imaging (MRI), diffusion-weighted imaging (DWI), and 1,356 large-format cellular resolution (1 mm/pixel) Nissl and immunohistochemistry anatomical plates (Fig 3G), and this is the initial atlas for the recently started BICAN consortium [5]. The atlas is annotated in 3D to over 150 structures (Fig 3H) and has been rereleased under a CC-BY-4.0 license in 2022 to support broader community use. This human atlas forms the starting anatomic context for the next BICAN phase.

Mapping brain data to reference spaces is challenging and uses a range of manual and automated methods of image registration (see BICCN image processing pipelines). Given the challenge of determining anatomical context for any reference atlas even within the mouse, precise image registration requires the whole brain image series (or a reasonable fraction of the brain) to be present with sufficient distinctive anatomical landmarks. Omics data often may not have detailed structural localization and can be positioned within a CCF through coordinate based, visual, or ontological tagging. Human data are typically of this type, where anatomic ontology is known and localized using annotated atlas plates and MNI space from the MRI reference brain volume (ICBM 2009b Nonlinear Symmetric; [35]. Mapping of these tissues is effectively done using the Cell Locator (RRID:SCR_019264)), developed in collaboration with Kitware (www.kitware.com). See S1 Text—Common Coordinate Frameworks for more information.

### The BICCN data ecosystem

**Fig 4:**
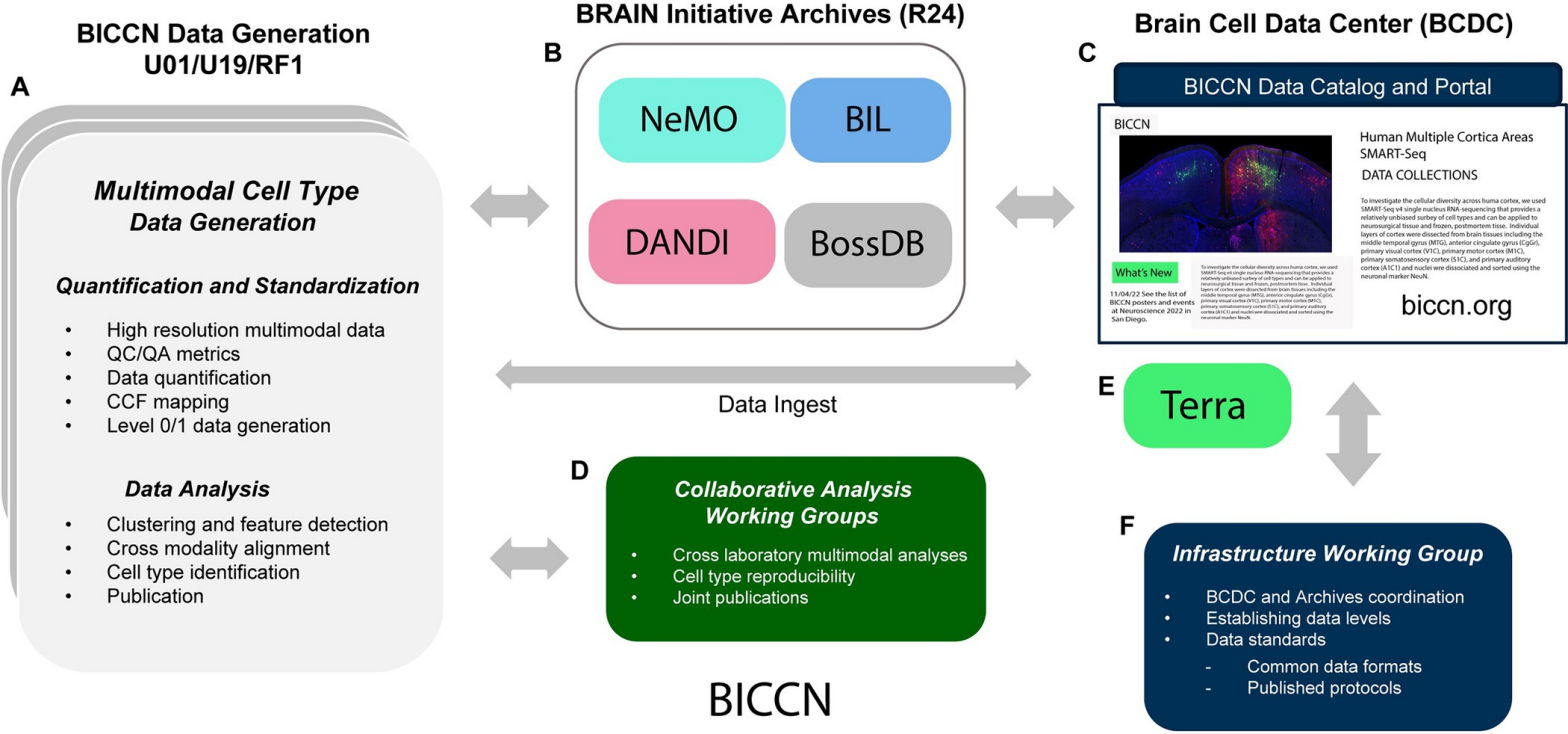
BICCN data ecosystem. **(**A) Multimodal cell type data generation by UM1/U01/19, RF1 centers produce high-resolution Level 1 multimodal data. (B) Data are submitted to one of 4 BRAIN archives depending on data type(s): Neuroscience Multi-Omic Data Archive (NeMO), Brain Imaging Library (BIL), Distributed Archives for Neurophysiology Data Integration (DANDI) for neurophysiology data, and Brain Observatory Storage Service and Database (BossDB) for electron microscopy ultrastructural datasets. Datasets are indexed and referenced (C) by the Brain Cell Data Center (BCDC; biccn.org), which provides a portal for accessing the consortium’s data, tools, and knowledge. (D) Laboratories engage in collaborative cross-modality interpretation of data and results. (E) Terra cloud-based platform for standardized omics processing accessible through BCDC. (F) An infrastructure working group oversees architectural development and workflow management.

The BICCN data workflow includes 3 distinct components, from work in individual centers, followed by ingestion and storage in dedicated archives, and ending in data catalog and portal in the BCDC (Fig 4). Multimodal data are generated by laboratories in multiple centers, which develop and apply robust methods for high-resolution, high-throughput mapping, including laboratory-specific QC/QA methods and data quantification (Fig 4A). Data analysis at individual laboratories is focused on rigorous signal detection and clustering, identification of modality-specific cell type taxonomies, and the validation of cross-modality associations. BICCN mandates broad and rapid data dissemination to accelerate scientific exploration and encourage community engagement, and all laboratories deposit Level 1 validated data quarterly to dedicated archives (Fig 4B). Finally, the BICCN data ecosystem is managed by the BCDC (Fig 4C). BCDC provides public access to and organization of the complex data, tools, and knowledge derived by BICCN, by supporting the acquisition of data from BICCN partners, providing data models and framework for importing structured data into the BCDC, and establishing semantic and spatial community standards for description and management of single-cell data modalities.

BICCN has a highly collaborative network for addressing multimodal analysis and cell type reproducibility across modalities and laboratories [7,36] (Fig 4D). Cross-institution analysis working groups tackle regional and whole brain analysis, which is facilitated by unrestricted access to quarterly released data to the archives. Systematic data processing provides a platform with common computational pipelines and environment for reproducible science across groups. For example, the BCDC provides access to Terra (https://terra.bio), a scalable and secure platform codeveloped by the Broad Institute, Microsoft, and Verily for biomedical researchers to share data and run analysis tools such as omics processing pipelines (Fig 4E). In addition, an Infrastructure and Standards Development group develops needed software, formalizes cross-modality standards, and specifies data structures, and protocols (Fig 4F) (see Standards and the BICCN: Towards FAIR Neuroscience.

**Fig 5:**
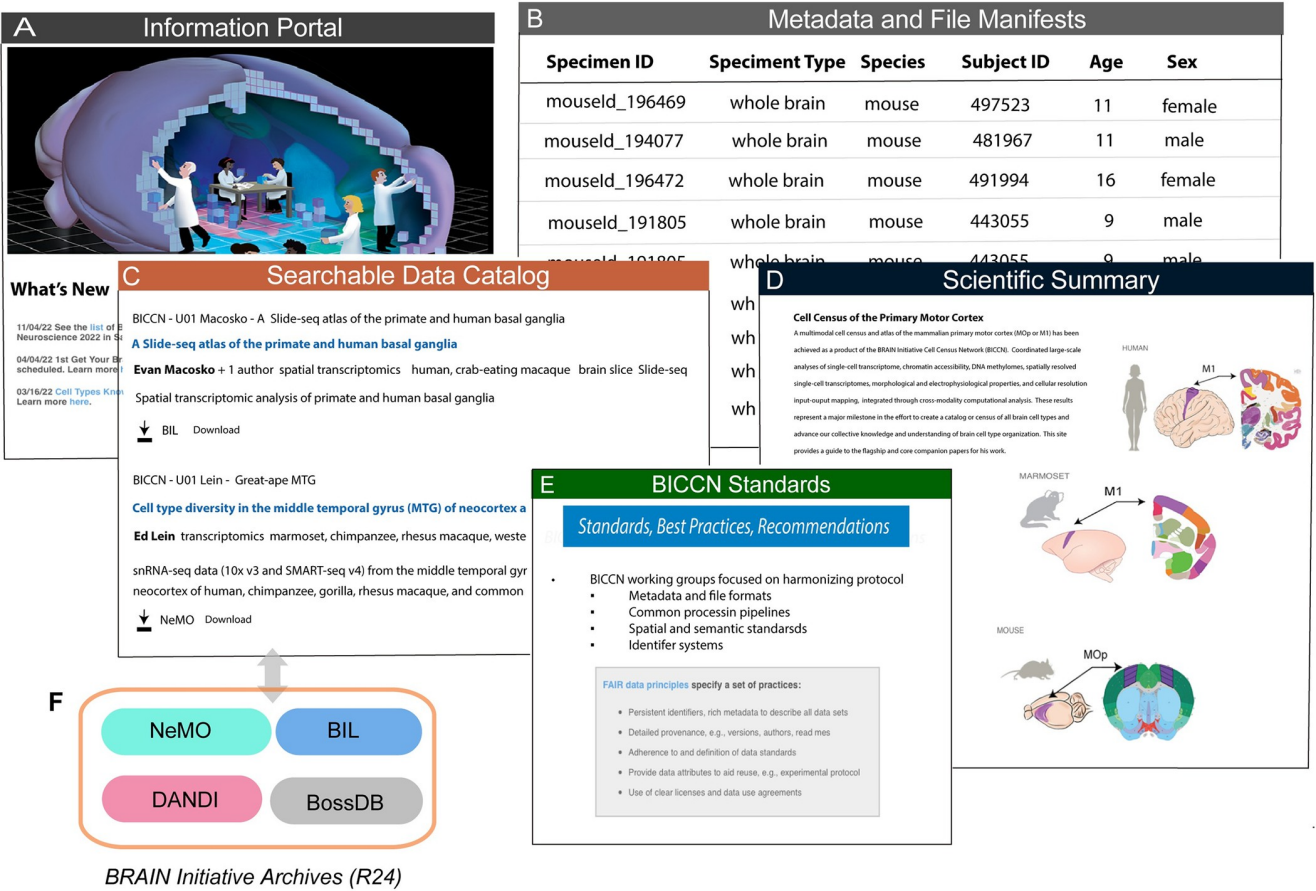
Brain Cell Data Center (BCDC). (A) The BCDC (www.biccn.org) supports the goals of the BRAIN Initiative Cell Census Network (BICCN) by providing a central public resource through the BICCN portal, which makes BICCN data and activities searchable from inside or outside the BICCN network. The portal includes (B) metadata and file manifests documenting data deposition from investigators into archives, (C) a searchable data catalog describing projects and datasets generated by the BICCN, (D) links to relevant publications with associated datasets and data mining tools, and (E) BICCN standards adopted by the consortium or created by internal working groups to ensure that data are harmonized across the consortium. (F) BRAIN Initiative archives are accessible from the BICCN data catalog. NeMO, Neuroscience Multi-Omic Data Archive; BIL, Brain Image Library; DANDI, Distributed Archives for Neurophysiology Data Integration; BossDB, Brain Observatory Storage Service and Database.

The BICCN Portal (www.biccn.org) is an entry point for BICCN resources and provides detailed investigator profiles, consortium news, data access, tools, standards documentation, policies, and overview of scientific progress (Fig 5A–5E). BCDC maintains a searchable data catalog listing all public datasets available through the BICCN portal. The BICCN catalog is built as an extension of the Allen Institute’s Brain Knowledge Platform and is currently accessible under the “Data access” tab at BICCN.org (https://biccn.org/data). The catalog organizes datasets by projects, each with one or more associated datasets that may be stored in a single archive or distributed across multiple archives (see Data archives for the BICCN). Users can browse the catalog or use a flexible search function (Fig 5C) to filter data by species, modality, techniques, and specimen type. For each dataset, the catalog provides basic descriptive metadata (Fig 5B), information on the dataset release status, the terms of the use, and a link to the location in the archive. Clicking on the link brings the user to a landing page that provides the dataset identifier, descriptive metadata, a download link, and additional relevant information.

### Data archives for the BICCN

BICCN data archives ensure that data are FAIR (See Standards and the BICCN: Towards FAIR Neuroscience), while optimizing storage costs to store and process data, enabling reproducible data practices, and effectively managing interchange between data producers and computational analysts. The archives serve as active repositories with corresponding.

Compute capabilities that enable collaboration within and across labs and serve as an entry point for research for all neuroscientists. Each archive supplies its own documentation on data submission, access, and reuse. There are currently 4 archives (of 7 BRAIN Initiative–supported archives) that are central to BICCN-related data types: Neuroscience Multi-Omic Data Archive (NeMO; https://nemoarchive.org), Brain Imaging Library (BIL; https://www.brainimagelibrary.org), Brain Observatory Storage Service and Database (BossDB; https://bossdb.org), and Distributed Archives for Neurophysiology Data Integration (DANDI; https://dandiarchive.org) (Fig 5F). The archives supply permanent and archival storage capabilities for transcriptomic and epigenomic data, imaging-based data including tracing, slice and whole brain morphology, density distribution, electrophysiology, and functional imaging, and ultraresolution electron microscopy (see Fig 2).

### Neuroscience Multi-Omic Archive

The NeMO archive (RRID:SCR_016152; https://nemoarchive.org) stores and disseminates omics data from the BRAIN Initiative and related brain research projects. NeMO stores both transcriptomic and epigenomic data, including transcription factor binding sites and other regulatory elements, histone modification profiles and chromatin accessibility, levels of cytosine modification, and genomic regions associated with brain abnormalities and disease. Data are organized by projects and within each project further organized by laboratory where data were generated, grant, organism, and the assay type. NeMO is consistent with the principles advanced by the NIH Strategic Plan for Data Science [37], including FAIR Principles, documentation of APIs, data-indexing systems, workflow sharing, use of shareable software pipelines, and storage on cloud-based systems.

Data archived at NeMO include raw sequence files as well as derived intermediate files such as BAM files (BAM | Integrative Genomics Viewer) and analyzed results including counts and cluster information (Data Levels 1 and 3), and metadata are submitted to BCDC. Sequence-level data for human samples submitted with access restrictions are made available through an approval process in conjunction with the NIMH Data Archive and NeMO archive. Data submissions rely on the fast transfer technology Aspera (https://www.nemoarchive.org/resources/aspera/). To upload data to NeMO, a user obtains credentials.

### Brain Image Library

The BIL (RRID:SCR_017272; https://www.brainimagelibrary.org) provides a persistent centralized repository for brain microscopy data and supports dataset deposition, integration into a searchable web-accessible system, redistribution, and analysis tools. It allows researchers to process datasets in-place and to share restricted and prerelease datasets. BIL includes whole-brain microscopy image datasets and their accompanying higher-level derived data such as neuron morphologies, targeted microscope-enabled experiments including connectivity between cells and spatial transcriptomics, and other historical collections of value to the community. In addition to the BICCN, BIL accepts all microscopy data relevant to the BRAIN Initiative, including data from primates, and most mammals and model organisms. BIL accepts both raw and processed data, and Data Levels 2 and 3, such as neuron tracings, which can be linked to lower-level data sources. While BIL does not limit the amount of data deposited per dataset or investigator, users planning to deposit more than 50 TB of data in a single year should contact in advance to discuss data deposition plans. Data contributed to BIL following the Standard metadata for 3D microscopy schema [38] are issued DOIs. Higher-level traced neuron data are accepted in the SWC format [37].

The BIL Analysis Ecosystem provides an integrated computational and visualization system to explore, visualize, and access BIL data without having to download it. Its Analysis Ecosystem provides large memory nodes, GPU nodes, and access to high-performance computing (HPC) resources for extensive data exploration. The Analysis Ecosystem virtual machine (VM) system has a remote desktop environment to run applications such as Fiji [39] and Vaa3d [40] and supports custom web gateways and commercial software. An Open-OnDemand gateway at BIL offers interactive access to popular scientific applications such as Jupyter Notebooks. A search portal provides pointers to the data on the BIL Analysis Ecosystem as well as download links. Finally, workshops are offered on a regular basis on how to interact with data through the BIL Analysis Ecosystem, the data submission process, and additional services [37].

### Distributed Archives for Neurophysiology Data Integration

The DANDI (RRID:SCR_017571; https://dandiarchive.org) is a web platform for scientists to share, collaborate, and process data from cellular neurophysiology experiments. DANDI works with BICCN and other BRAIN Initiative groups to curate data using community data standards such as Neurodata without Borders (NWB; [41]) and Brain Imaging Data Structure (BIDS; [42]) and to make data and software for cellular neurophysiology FAIR. Currently housing nearly 500 TB of data across 6 species and multiple instruments and techniques, the DANDI archive stores, publishes, and disseminates neurophysiology data including electrophysiology, optical physiology, and behavioral time-series, and images (MRI and microscopy) from immunostaining experiments.

DANDI datasets are referred to as Dandisets and include the dataset and file metadata. Supplied per-file metadata includes instrument, species, sample, subject, and other experimental details. Each Dandiset is organized in a structured manner to help users and software tools interact with it and has a unique persistent identifier that can be used for citation. The DANDI web application allows users to browse and search for Dandisets, create an account to register a new Dandiset or gain access to the Dandi Hub analysis platform, add collaborators to a Dandiset, and retrieve an API key to perform data upload. DANDI has enabled streaming access to parts of data using a combination of cloud technologies and storage formats, allowing for more scalable analysis software and visualization technologies. DANDI exposes all data as versioned DataLad datasets [43], allowing users to overview an entire dataset without downloading any data to their local file system and then to selectively download specific files or folders. DANDI provides a programmable interface to the archive and Jupyter computational environment, and an API allows development of other software tools for accessing, searching, and interacting with the data in the archive.

### BICCN data processing pipelines

The BICCN ecosystem includes production-level, cloud-native data processing pipelines, developed by the Broad Institute’s Data Sciences Platform (DSP) in collaboration with BCDC. While BICCN investigators and other users often process their own omics datasets, standardized pipelines are used to supplement and integrate original analyses with uniformly processed datasets. The pipelines leverage consistent standard file schema and types as well as standardized quality control metrics and metadata. The established cloud-native pipelines replicate processing used by several consortium groups including computational pipelines for processing single-cell/nucleus 10× v2/3, sc/sn full transcript, sn-ATAC-seq, and snmC-seq sequencing data. Each of these pipelines was developed in collaboration with a sponsoring BICCN group and captures their expertise in data processing (Table 1; for additional pipeline documentation, type “BICCN” in the WARP Documentation search bar (https://broadinstitute.github.io/warp/docs/get-started). Detailed documentation and user guides are available through www.biccn.org. Workflow Description Language (WDL) Analysis Research Pipelines (WARP WDL Code) repository contains a collection of cloud-optimized pipelines.

**Table 1:** BICCN molecular pipelines.

Pipeline	WARP WDL Code	Input Data	Overview	Terra Workspace
Smart-seq2 Single Nucleus Multi-Sample (RRID:SCR_021312)	Smart-seq2 Single Nucleus Multi-Sample	Single-cell data generated with Smart-seq2 assays	Smart-seq2 Single Nucleus Multi-Sample Overview	Smart-seq2 Single Nucleus Multi-Sample
Optimus (RRID:SCR_018908)	Optimus	10× Genomics v2, v3 3’ single-cell and single-nucleus data	Optimus Overview	Optimus
Single-Cell ATAC-seq (RRID:SCR_018919)	scATAC	Single-cell ATAC-seq data from nuclear isolates	scATAC Overview	scATAC
MethylC-Seq (RRIS:SCR_021219)	CEMBA	Multiplexed single-nucleus bisulfite sequencing data	CEMBA Overview	CEMBA

The Broad Institute Data Sciences Platform resources are actively used by other individuals and consortia, and the approach to the development of molecular pipelines for the BICCN is inspired by FAIR principles [18]. This includes use of Research Resource Identifiers (RRIDs) to give pipelines unique, explicit identifiers and host the pipelines in multiple community resources including public GitHub repositories (for software engineers), Dockstore (https://dockstore.org) for computational biologists, and Terra (where the pipelines are preconfigured and ready to run for those without local infrastructure or who want to use scalable cloud resources). Use of a modern WDL separates the code performing scientific tasks from code orchestrating the pipeline on infrastructure and encouraging interoperability for reproducible science (see Section Standards and the BICCN: Towards FAIR Neuroscience.)

### BICCN image processing pipelines

Image registration, mapping, and alignment are necessary to bring data from individual brain samples into common coordinate systems, yet often challenging to standardize. The choice of which software to use often depends on the computational resources available, integration with other image tools (e.g., visualization, neuron reconstruction), and which algorithm is most effective for the image data at hand. Two image processing platforms were developed or extended through the BICCN data ecosystem. (1) Generative Diffeomorphic Mapping (GDM) for image registration and atlas mapping from the Brain Architecture Portal (http://brainarchitecture.org) combines multimodal imaging datasets such as ex vivo radiology and histology in the same animal/subject. The GDM approach overcomes challenges in registering tissue processing procedures such as extraction and fixation that cause brain tissue deformation (S1 Text—CCF Mapping). (2) The Image and Multi-Morphology Pipeline [40,44] accesses raw images from the BIL archive and implements the full pipeline of conversion, processing, morphometry generation, registration and mapping, release, and analysis. This pipeline is hosted on an open cloud platform that features collaborative processing and synergetic computing among various clients, and web interfaces. All data on the server can be accessed through MorphoHub [45], a petabyte-scale multimorphometry management system and integrates the 3 largest whole-brain full morphology datasets [28], MouseLight [46], and single-neuron projectome of mouse prefrontal cortex [46,47] (S1 Text—Image Processing Pipelines).

While not all BICCN image data are mapped into the CCFv3, a wide variety of tools were improved through BICCN collaboration (Fig B in S1 Text). These image registration packages include ANTs, which maximizes image cross-correlation while ensuring that maps between images are smooth and invertible [48], Elastix, whose modular design allows users to compare different registration algorithms [49], and 3D Slicer, which offers both landmark and grayscale image-based registration [50]. Highly flexible registration tools such as QuickNII and VisuAlign directly map to the CCFv3 and focus on registering high-resolution 2D images [51]; CloudReg, which is a cloud-compliant pipeline for intensity correction, image stitching, and diffeomorphism-based registration [52]; and mBrainAligner [44], which is cross-modal and integrates with the Vaa3D software suite and can also be freely accessed through the web server mBrainAligner-Web [33,44]. Additional cloud-based Petabyte data generation and management system MorphoHub [45] was also developed to assist additional data analysis. These platforms are developed through open-source and extensible approaches, are accessible to the public, and can be extended through plugins (S4 Table).

### Working with BICCN data

The BICCN has developed many tools and applications to work with BICCN data. An inventory of these tools describing their application to single-cell analysis is provided under the “Tools and analysis” tab of the BICCN portal Tools and Analysis - Brain Cell Data Center (BCDC). Some of these resources are described below, with an emphasis on those that facilitate integrative analysis.

### Cell Type Knowledge Explorer

The Cell Type Knowledge Explorer (CTKE; RRID:SCR_022793) is an interactive application that aggregates multimodal BICCN data from the primary motor cortex (MOp) atlas at the level of individual cell types in mouse, human, and marmoset. The CTKE integrates the work of many BICCN laboratories and presents aggregate knowledge about cell types in the form of data visualizations and text summaries. Drawing inspiration from Gene Cards (genecards.org; [53]), information is displayed on over 400 individual panels across the 3 species. The CTKE is powered by a data-driven ontology [54] linking MOp atlas data to a well-established body of knowledge on neurobiology enabling text-based search of the data by cell type names, minimal sets of marker genes from the NS-Forest algorithm [55], and historical terms from the literature (“pyramidal,” “chandelier,” etc.).

**Fig 6:**
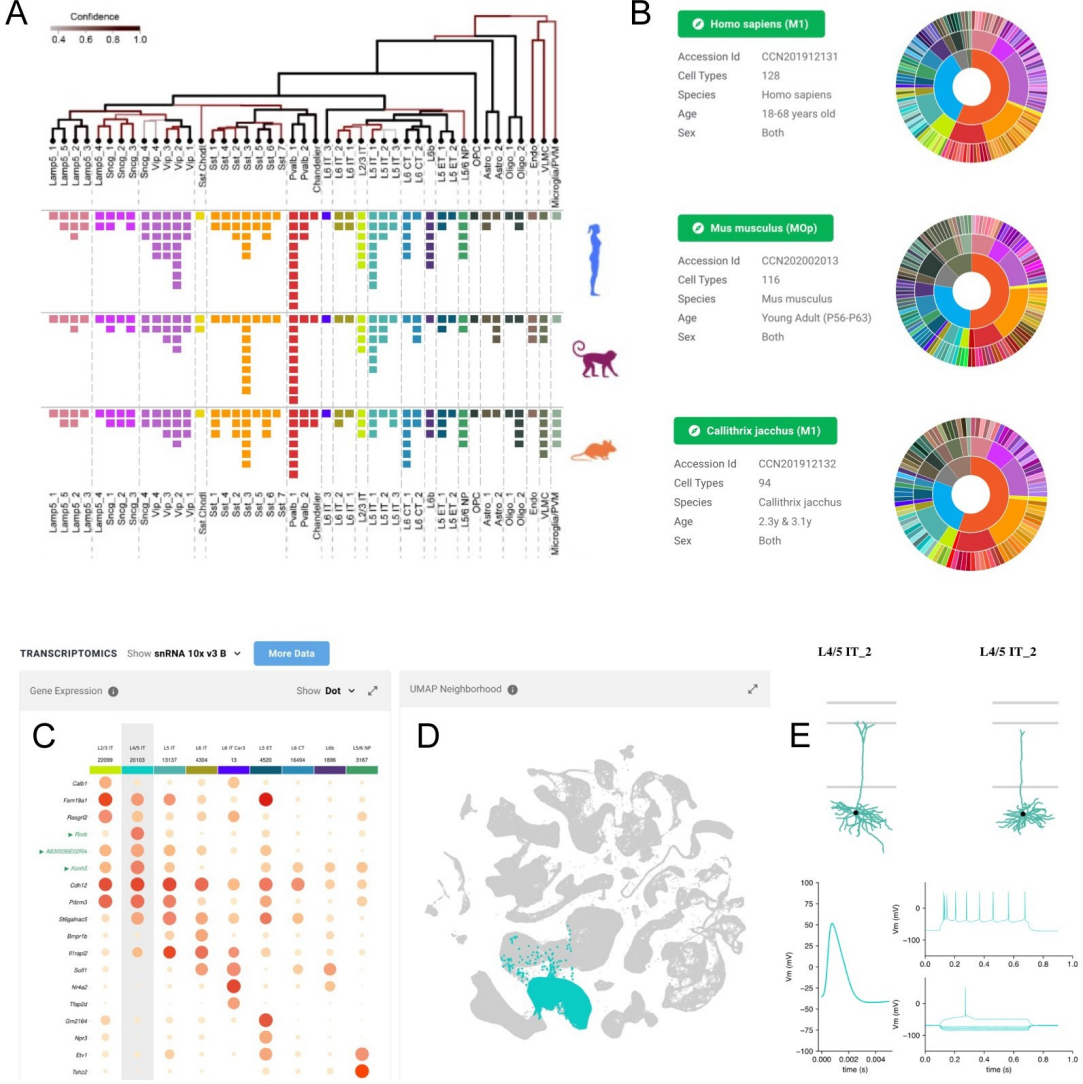
Cell Type Knowledge Explorer. CTKE is an interactive tool that aggregates multimodal BICCN data from the primary motor cortex mini atlas at the level of individual cell types in mouse, human, and marmoset. (A) Cross-species aligned taxonomies and common cell types in MOp, (B) cell types in each species are accessible and linked through interactive sunburst plots, (C) marker gene panels defining the L4 cell type in mouse, including machine learning–based NS-Forest markers [55] highlighted in green, and (D) rendering of expressing cells in UMAP, (E) morphology and electrophysiology exemplars associated with cell type.

By leveraging BICCN’s cross-modality mapping of nontranscriptomic data to expression-based taxonomies, CTKE provides rich phenotypic information about cell types and enables its systematic exploration. Cell Type Knowledge Cards for each of the 3 taxonomies are accessible from an interactive sunburst plot (Fig 6A and 6B). Each card visually presents the molecular signatures of cell types derived from single-cell transcriptomics and may also include morphological reconstructions, exemplar action potential traces, and summaries of electrophysiological characteristics, or spatial locations determined using spatial transcriptomics. Genome browser views show accessible chromatin data at marker gene locations and predicted cell type–specific enhancer regions and links to homologous cell types across species. These modalities are represented on a given card in unique panels, each of which includes links to reusable source data and additional BICCN visualization and analysis tools.

CTKE also helps researchers to annotate and interpret their own data. For example, CTKE includes links to Azimuth [56], a web application that provides utilities to map single-cell expression data to curated reference datasets. This allows users to derive cell type annotations for their own datasets in the context of BICCN primary motor cortex mini atlas data for the human and mouse. Similarly, CTKE facilitates the interpretation and annotation of other data types. For example, a researcher studying mouse MOp may have immunohistochemistry data indicating that the gene *Rorb* is highly expressed in a certain population of cells and want more information about what type of cells they might be. Searching “*Rorb*.*”*

In the CTKE would return the L4/5 IT neuron subclass as a cell type that expresses *Rorb* more highly than other MOp types (Fig 6C and 6D, “Transcriptomics” panel). Navigating to the “Spatial Transcriptomics” and “Morphology” panels would reveal that L4/5 IT neurons are found at a similar cortical depth and with similar morphological characteristics to those this researcher sees in their cell population of interest (Fig 6E). If this researcher were interested in understanding whether these cell types are present in humans, they could navigate to the “Cross-Species Cell Types” panel on the Cell Type Knowledge Card for the L4/5 IT_1 subtype, where they would also find several putatively homologous types and be able to navigate directly to their cards for further investigation. In summary, the CTKE strives to provide a user-friendly interface for deep exploration of the BICCN primary motor cortex mini-atlas in a cell type–centric manner and provides a framework for extending to other brain regions and future data navigation tools for whole-brain multimodal atlases.

### NeMO Analytics

**Fig 7:**
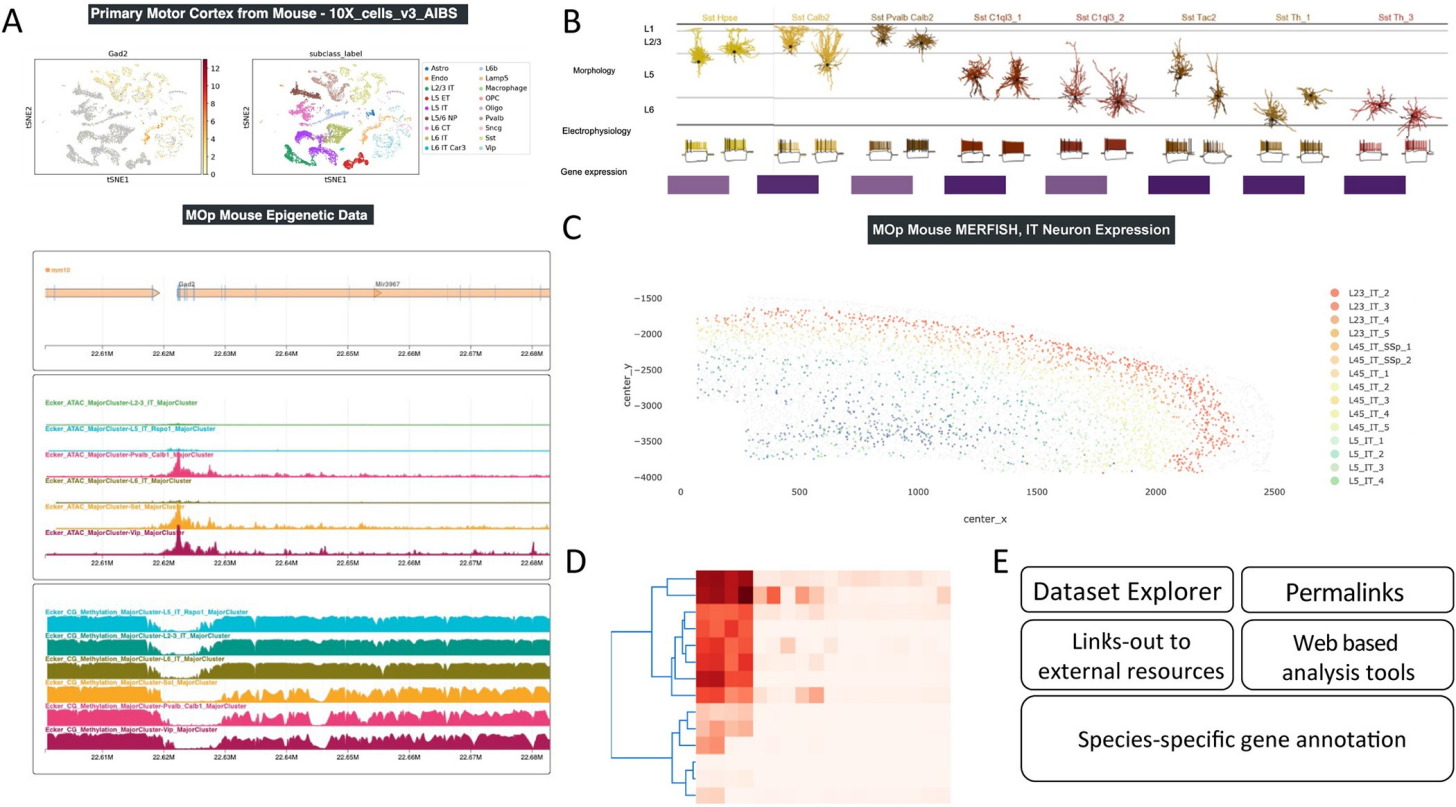
Analyzing BICCN datasets with NeMO Analytics. NeMO Analytics provides direct access to many of the BICCN multiomic datasets for comparative analysis, visualization, and data mining. (A) Example NeMO Analytics profile showing glutamic acid decarboxylase 2 (*Gad2*) expression and epigenetic changes in the datasets of [8]. This profile can be found at NeMO Analytics. (B) Integrated visualization of Patch-seq morphology, electrophysiology, and gene expression for cell types in primary motor cortex NeMO Analytics [13]. (C) Visualization from a MERFISH experiment with spatial distribution of cell types NeMO Analytics [12]. (D) NeMO Analytics offers a variety of web-based visualization and analysis tools including heatmaps, volcano plots, and a single-cell workbench allowing for de novo analysis of datasets. (E) Additional utilities of NeMO Analytics.

NeMO Analytics (RRID:SCR_018164; https://nemoanalytics.org) is a web-based suite of data visualization and analysis tools for single-cell data analysis. The portal allows users to explore single-cell, single-nucleus, and spatial transcriptomic and epigenetic profiling data, with flexible plotting tools allowing side-by-side comparisons of any data type. The portal is prepopulated with thematically organized datasets reflecting projects across BICCN. Users can upload their own data for private or public use, utilize curated datasets from other users, select a dataset from the NeMO Archive, or benefit from data collections hosted from peer-reviewed publications. NeMO Analytics simplifies access to BICCN data and provides nonprogrammers with a suite of analytical tools for data exploration, including cell cluster visualization based on expression/cell type, cell cluster comparison, identification of marker genes across datasets, plotting multigene analyses (e.g., heat maps, volcano plots, violin plots for groups of genes), and note taking (Fig 7). Additional tools include a workbench to perform de novo analysis of scRNA-seq data, visualization and analysis of spatial transcriptomics data, and visualization of epigenomic data. The platform supports visualization of datasets across species and modalities side by side and linked by homologous gene symbols. The links to the datasets in NeMO Analytics are embedded in the figure legends, providing seamless access to the data.

### Mouse Connectome Project

**Fig 8:**
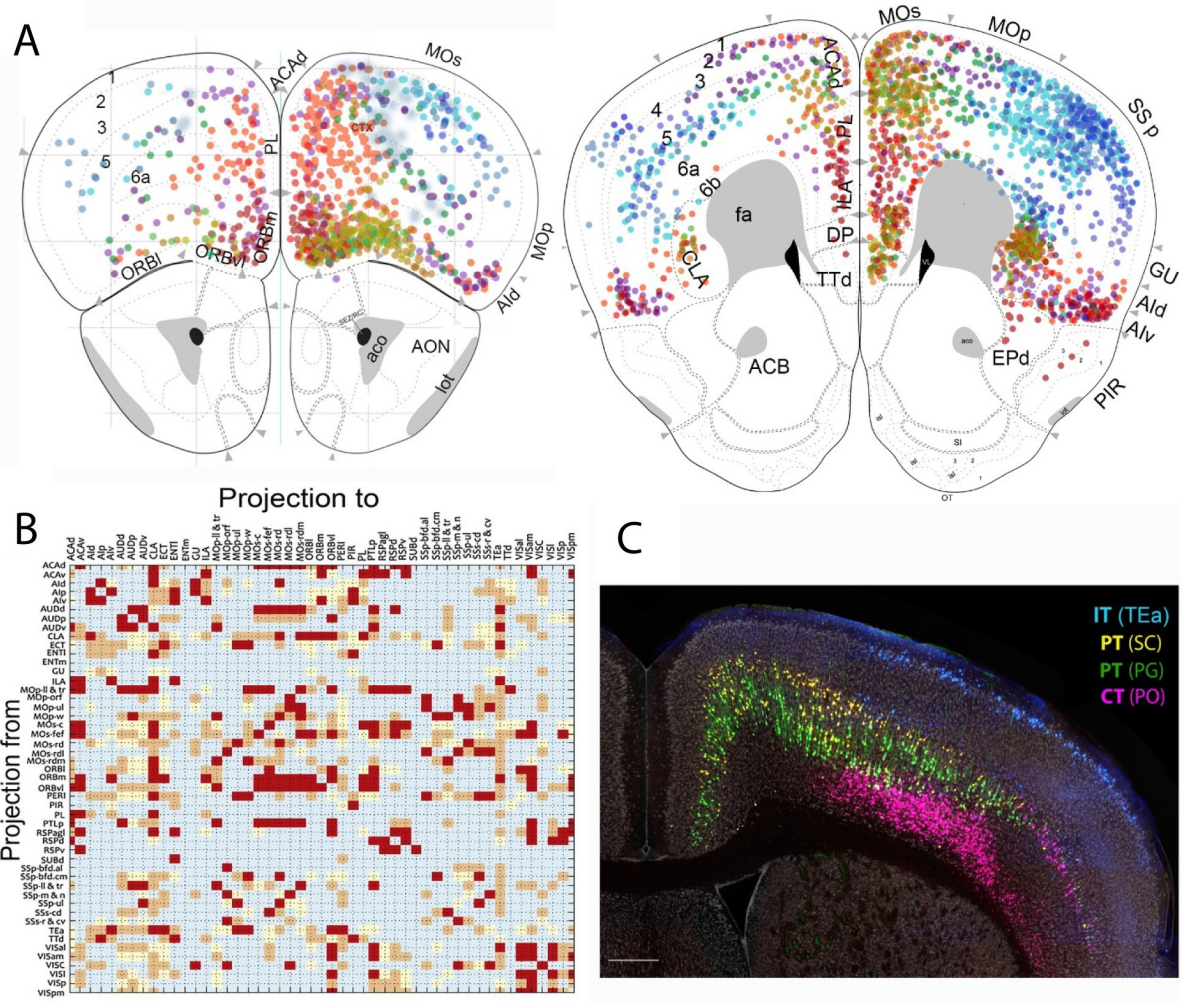
Neuronal Connectivity and Mouse Connectome Project (MCP). **(**A) Connectivity map of distinct cortical projection neurons (cell types) in the prefrontal cortex. **(B)** A connectivity matrix constructed based on these retrograde tracing data. These data resources are available on iConnectome (www.MouseConnectome.org). (**C**) Example of back labeled neurons in the cortex following injections of 4 retrograde tracers into the temporal association area (TEa), superior colliculus (SC), periaqueductal gray (PG), and posterior thalamic nucleus (PO), revealing 3 major classes of cortical neuron types, IT, PT, and CT. Scale bar = 250 μm.

The Mouse Connectome Project (MCP; RRID:SCR_004096; https://cic.ini.usc.edu; https://brain.neurobio.ucla.edu/) has systematically produced and collected connectivity data for over 10,000 neural pathways in >4,000 experimental cases utilizing a variety of multifluorescent pathway tracing techniques that included double coinjections, triple anterograde and quadruple retrograde tracing, Cre-dependent double AAV anterograde tracing, and rabies viral-based Cre-dependent retrograde methods [57,58] (Fig 8). This combination of injection strategies can [59] simultaneously reveal key connectivity information for a given brain region and enables construction of detailed connectivity maps and to systematically assemble neural networks of different functional systems in the mouse brain. Complementary to the molecular cell typing strategies described above, these connectivity data provide a fundamental framework for cataloging neuronal types based on anatomic locations, projection targets, and morphological features (Fig 8A). In each animal, up to 4 retrograde tracers are injected into different cortical locations to retrogradely label all neurons that send projections to the injected areas. Because the injections collectively span the entire neocortex, theoretically, for any given cortical area, all neuronal populations (corticocortical projection neurons) that innervate different cortical targets are demonstrated. Distributions of these retrogradely labeled neurons were annotated to construct a connectivity matrix to visualize corticocortical network organization and a connectivity map to enable direct comparisons of regional and laminar-specific distribution patterns of neuronal populations (cell types) associated with each cortical area (Fig 8C); see also www.MouseConnectome.org/Corticalmap). Multiple retrograde tracer injections into different cortical (i.e., temporal association area) and subcortical areas (i.e., the superior colliculus, periaqueductal gray, posterior thalamic nucleus) simultaneously reveal multiple cell types, namely, intratelencephalic (IT), pyramidal tract (PT), and corticothalamic projecting (CT) neurons (Fig 8C). This connectivity map and derived catalog of anatomically defined neuron types provide complementary and confirmatory information for molecularly defined neuron types described in other BICCN resources [8,15]. Finally, these multifluorescent retrograde tracing data are available through iConnectome (www.MouseConnectome.org; https://brain.neurobio.ucla.edu/maps/).

### Brain Architecture Project

A collection of high-resolution 2D images from BICCN collaborators are available through the Brain Architecture Project (BAP; RRID:SCR_004283; http://brainarchitecture.org) on the Brain Architecture web portal. Datasets are divided into species and experiment-type specific pages, accessible from the front landing page. Users can filter mouse cell distribution datasets via free text search of metadata for keywords and mouse projection and connectivity datasets via injection region or tracer. A high-resolution viewer capability to display overlays of regional compartments, points indicating cell bodies post-cell detection, and skeletonization on 2D sections of atlas mapped brains. The viewer can display data at multiple resolutions, with zoom to super-resolution capability, beyond the native in-plane 0.46-μm resolution of the images. All software employed for image analytics pipeline, including registration and atlas mapping, cell detection, and process detection and skeletonization via are available both in interactive versions on the Brain Architecture web portal, and for download (of both source code and documentation) on GitHub and Bitbucket repositories. There are additionally cross-linkages with the Broad Institute Single-Cell Portal (https://singlecell.broadinstitute.org/single_cell).

### Additional tools and resources

Numerous other resources have been key in analysis of BICCN publication datasets, described in S1 Text—BICCN Tools and Resources, S4 Table, and at https://biccn.org/tools. Tools key to BICCN publications are Epiviz (RRID:SCR_022796) and Brain Cell Methylation Viewer (RRID:SCR_020954), interactive visualization tools for functional genomics data; Brainome (RRID:SCR_018162), a genome browser to visualize the cell type–specific transcriptomes and epigenomes of cell types from the mouse MOp; Catlas (RRID:SCR_018690), which provides maps of accessible chromatin in the adult mouse isocortex, olfactory bulb, hippocampus, and cerebral nuclei; Cytosplore Viewer (RRID:SCR_018330) is a stand-alone application (Windows and MacOS) for interactive visual exploration of multispecies and cross-omics single-cell data in several BICCN data resources; and MetaNeighbor [60,61], a method for assessing the replicability of single-cell data, used in a number of key BICCN publications (e.g., [8,9]) to validate cell types and perform quality control. An important resource for the analysis of single-cell brain data is the Broad Institute Single Cell Portal (RRID:SCR_014816).

Several open-access neuroinformatics resources launched prior to BICCN efforts [62] but were substantially expanded with support and contributions from BICCN projects and have been utilized in multiple BICCN publications. Two such examples, NeuroMorpho.Org (RRID:SCR_002145) and Hippocampome.org (RRID:SCR_009023), have helped bridge seminal literature information and data with new BICCN-generated data. NeuroMorpho.Org [63] provides free access to hundreds of thousands of reconstructed neural cell morphologies contributed by over 900 laboratories worldwide from approximately 100 distinct species and were utilized in the recent comparative analysis of neocortical neurons [9], where BICCN data from human, marmoset, and mouse were augmented with tracings from other mammals. Hippocampome.org [64] is a knowledge base of neuron types from the mammalian hippocampal formation and entorhinal cortex with more than 500,000 neuronal properties extracted from 46,000 pieces of evidence annotated from scientific articles. For more details on the above and additional resources, we refer readers to the BICCN online resource Tools and Analysis - Brain Cell Data Center (BCDC).

### Standards and the BICCN: Towards FAIR Neuroscience

To be reused and shared efficiently, accessible data need to be described in standard ways, and the development and adoption of standards is thus essential to advancing rigorous science and efficient collaboration [65]. An increasingly comprehensive and detailed set of technical, quality control and policy standards developed or utilized by the BICCN provides guidance/best practices for consortia members and others seeking to use BICCN data. The BICCN is committed to implementing practices and technologies to make data and other research products FAIR [18]. All data that do not involve protected health information are made available under a CC-BY 4.0 attribution license [6].

BICCN Standards, Best Practices, and Recommendations have been implemented across BCDC and the BRAIN data archives including metadata and file formats, common processing pipelines, spatial and semantic standards, and identifier systems. BICCN Working Groups focused on harmonizing protocols, data formats, and metadata for transcriptomic, physiological, and anatomical data types. The BCDC coordinated the formation of working groups of consortium members that considered what standards and best practices were necessary for new experimental technologies for which standards were not yet available, including developing QC criteria for a given modality. The BCDC was also responsible for developing strategies to harmonize common metadata across the archives, including submissions checklists, collections metadata, and basic descriptive information for specimens. The Metadata and Infrastructure Working Group (Fig 4F), comprising representatives from the BCDC, the 4 BRAIN Archives housing BICCN data and BICCN investigators, coordinated the adoption and development of the necessary technical standards to support FAIR data. However, beyond basic descriptive metadata such as modality or species, annotations, and mappings at a deeper level are still nascent [66].

Additional standards were adopted over the course of the project as they became available, e.g., the Essential Metadata for 3D Microscopy standard developed with support from the BRAIN Initiative [38] was recently implemented by BIL. The independent data generation within the BICCN allowed post hoc assessment of standards for rigor and reproducibility meta-analysis. This is particularly true of the mouse expression data, which involved replicates across technologies and allowed assessment and integration to assess the replicability of cell type calling via, e.g., MetaNeighbor [60] and post hoc integration to produce more reliable marker sets [9,67]. BICCN-developed standards are available through a public GitHub Repository (https://github.com/BICCN) and BICCN Standards, Best Practices, and Recommendations - Brain Cell Data Center (BCDC).

### BICCN FAIR data practices

The BCDC, in partnership with the archives that house the data, ensures that all BICCN data are FAIR according to the principles set out in [18]. The BICCN ecosystem benefits and derives increased utility from the set of 15 FAIR data principles and recommendations. Although full implementation of FAIR was challenging, particularly in the initial phase of the BICCN where the archives, techniques, and standards were under simultaneous development, the BICCN has been moving toward implementation of a consistent set of baseline FAIR practices over the course of the project. The BICCN ecosystem benefits and derives increased utility from the FAIR data principles and recommendations. Summarized in Box 1 are the main areas where the BICCN data ecosystem has implemented these practices. This standards-based work includes use of persistent identifiers and rich metadata, detailed provenance, use of FAIR vocabulary, and use of clear data use agreements.

**Box 1. FAIR Neuroscience data practices and the BICCN**

**1. Use of persistent identifiers and rich metadata to describe all datasets**

- BICCN datasets receive a DOI or an equivalent persistent identifier from the archives;
- The BCDC and archives coordinate on standard metadata to accompany all datasets;
- Archives implement dataset landing pages for machine-readable rich metadata about the datasets and access.

**2. Providing detailed provenance**

- BICCN datasets are versioned;
- Full-citation metadata is supplied to support data citation;
- Investigators encouraged to link datasets to detailed experimental protocols deposited in at BICCN group at Protocols.io.

**3. Adherence to and definition of data standards**

- Archives are implementing community data standards, including those developed through the US BRAIN Initiative;
- Archives have implemented common file format and metadata requirements for specific data types;
- Standards in use in BICCN are documented at biccn.org;
- Several archives make use of standard identifier schemes for entities linked to the data such as ORCIDS for authors and RRIDs for organisms, antibodies, cell lines, and tools.

**4. Use of FAIR vocabulary**

- BICCN has developed ontologies and controlled vocabularies to annotate data and map metadata such as the Brain Data Standards Ontology;
- Vocabularies are all maintained in GitHub repositories as described on the BICCN standards page.

**5. Providing a plurality of data attributes to aid in reuse**

- Checklists for standard metadata for experimental types such as Patch-seq and for describing specimens,
- Contact person identified to answer questions about the data, and code that can be used with the data.

**6. The use of clear licenses and data use agreements**

- All data that do not involve protected health information are made available under a CC-BY 4.0 attribution license;
- BICCN requires that those using the data follow formal citation principles for citing the data;
- Archives are making citations available per dataset to assist in proper citation.

### The Brain Data Standards Ontology

An important component of cell type classification is a rigorous and precise ontology and nomenclature. The Brain Data Standards Ontology (BDSO) [54] is an ontology of cell types defined in the BICCN MOp that extends the Cell Ontology (CL) [68] to provide a more detailed set of terms for FAIR-compliant annotation than previously available. As an extension of CL, BDSO is fully interoperable with both CL and Uberon [69], allowing data annotated with BDSO terms to be interoperable not only with the BICCN data, but also with datasets from the wider community. This approach is scalable and lowers human error (compared to manually creating the ontology), allowing features that are crucial in scaling to whole brain annotation. As part of creating BDSO, representation of neuronal cell types in CL has been deepened, adding new cortical cell types by defined markers, projection pattern (e.g., extratelencephalic projecting), layer, and morphology (e.g., pyramidal). These additions to CL have already been used for annotation in datasets in CellXGene [37], the Cell Annotation Platform (RRID:SCR_022797), and other single-cell transcriptomics data providers to deepen annotations to use terms from BDSO. A major application of BDSO is to support organization, navigation, and searching of data in the CTKE. Knowledge graphs and APIs were developed for the CTKE (Knowledge graph: http://purl.obolibrary.org/obo/pcl/bds/kg/; API: http://purl.obolibrary.org/obo/pcl/bds/api/), making the reuse, search, and navigation of the BDSO openly accessible. The latest release of the ontology is hosted at BDSO (S1 Text—Brain Data Standards Ontology).

### From BICCN to the BRAIN Initiative Cell Atlas Network (BICAN)

Advances in the development of laboratory techniques and analysis methods for single-cell data in the mammalian brain has made feasible a characterization of its fundamental cell types. Beginning with 10 pilot studies [2] in developing, validating, and scaling up emerging genomic and anatomical mapping technologies, the BICCN has used these approaches toward generation of complete, accurate, and permanent (CAP) data resources to form an extensive data ecosystem. The BICCN has completed cell type profiling using transcriptomics (10× RNA-seq) and epigenomics (ATAC-seq) for the whole mouse brain, and in many regions of the human brain, and is developing architecture, infrastructure, and product resources to support these data. In addition to ongoing BICCN datasets produced by individual laboratories and resulting publications, 6 active BICCN Working Groups are presently engaged in continuing collaborative projects (BICCN 2.0) integrating and interpreting new and existing data. In addition to fulfilling the goal of integrating transcriptomic and epigenomic data across the entire mouse central nervous system, these groups are developing methods to identify cell type–specific enhancers that can drive systemic delivery of reporter genes to select subclasses or types of brain cells in mice and primates, producing molecularly annotated wiring diagrams of the mammalian brain, and now beginning work on developing comprehensive human and NHP atlases, through the recently launched BICAN. Additional work is in measuring proteomic signatures of brain cells and further developing integration methods and infrastructure for future atlases.

While significant progress has been made in most aspects of the original BICCN infrastructure vision, much work remains and is continuing with new BICAN activities. The resources developed through the BICCN have been the result of active collaborations between data generators, analysts, informaticians, and software developers, and the ultimately desired data ecosystem will support data collection, quantification, and a mapping framework for managing data and information across diverse repositories. This ecoystem should maintain consistent data description standards that describe and facilitate best FAIR practices for community use of multimodal single-cell data and its content. From early in the consortium’s activities, requirements for FAIR data management were identified; however, the goal of building a foundational community resource for housing single-cell centered data content in the brain is still a work in progress.

An important component of full data integration is the spatial mapping of data enabling users to search by spatial location for data of interest, and common coordinate frameworks for mapping must be in place. There has been general community acceptance of the Allen Mouse Brain Common Coordinate Framework (CCFv3) with several tools now available for pinning specimen level or registering and aligning spatial sections or volumes. Components are now in place for a fully searchable and spatially resolved database, although there remains engineering work to incorporate these into a functional application. Gaps also remain in the BICCN infrastructure. The BCDC data catalog offers an entry point to each project and dataset, yet specimen-level search and access enumerating regional or nuclei-level search is not at present universally available. Further, while the data archives are all capable of accepting and managing large data volumes, and many tools are available for accessing relevant data, the workflow is not yet fully interoperable and there are still inconsistent metadata standards across modalities. This is particularly challenging for users of multimodality data types such as Patch-seq where the associated data types, transcriptomic, morphology, and electrophysiology are stored in different archives.

BICCN data represent unprecedented coverage describing the cell type landscape of the mammalian brain, and the stage is now set for completing the BRAIN Initiative 2025 [37] vision of large-scale profiling of the human brain including diversity and development. This new phase, commenced in Fall 2022, is the BICAN [5] and is the extension of the groundwork set by the BICCN. The extension of BICCN to BICAN is essential to understand which cell types are unique to humans and to identify precise relationships with cell types of the mouse and NHP. BICAN presents novel challenges of human tissue management and sample selection, the need for improved standardization of sequencing and mapping, and establishment of a more integrated neuroinformatics framework. BICAN will also present major challenges in establishing standard protocols, mapping, and annotation, but much work can be leveraged from BICCN ecosystem. The neuroinformatics work of the BICAN initiative calls for standardized sequencing and tissue selection, and for the creation of an integrated knowledge base for the community [70].

The ultimate expectation of BRAIN 2025 is to accomplish a full census of neuronal and glial cell types in mouse, human, and NHP, an intellectual framework for cell type classification, and to provide experimental access to the different brain cell types to determine their roles in health and disease. However, there is not yet full consensus on what a neuronal type is, since a variety of factors including experience, connectivity, and neuromodulators can diversify the molecular, electrical, and structural properties of initially similar neurons. There is also increasing evidence that there may not even be sharp boundaries separating subtypes from each other, and cell phenotypes may change over time. Here, taxonomies of putative types and representative cells will provide a frame of reference for studies across labs, and possibly in different organisms, allowing cross-comparison. The data and resources under development in the combined BICCN/BICAN data ecosystems should provide researchers with tools to address these challenges.

The extension of the multimodal cell-type atlas of select regions in the human brain to multiple brain regions, particularly those housing vulnerable cell populations, and to different stages of brain development is essential. Such openly available datasets will be key to future studies comparing cell types within their spatial context in the normative brain to those in neuropsychiatric disease, with the addition of transcriptional, epigenetic, morphological, and neurophysiological datasets from postmortem brains, either within the BICCN and BICAN data archives or published in the literature. Combining the BICCN and BICAN data archives with the ability to place cells within a CCF detected from deidentified digital pathology data will also make available large datasets that provide the sample numbers and diverse representation necessary for use of interpretative machine learning analysis applications. Moreover, as techniques for spatial detection of proteins and metabolites achieve multiplexing capabilities as well as cellular resolution, such data may help to uncover disease mechanisms that may be beyond transcriptional and epigenetic detection but, when combined with data currently included in the BICCN datasets, could help explain the neurophysiological changes detected in specific cell types and brain areas as part of a disease phenotype.

The BICCN has provided the community with massive high-quality datasets describing the multimodal cell type landscape of the mammalian brain. Substantial resources now exist for the study of brain cell types, and while the supporting data ecosystem is not yet complete, tremendous progress has been made. Increasingly diverse skills are being applied to the architectural design and development of the new BICAN data ecosystem, and we are planning for continuous extension and enhancement of this work to address human-specific challenges. We are only beginning to interpret this valuable data and to understand its importance for the nature of cell types in the brain.

## Supporting information

S1 Table BICCN grant awards, data modalities, techniques, and species profiled. BICCN Grants: Lists all BICCN investigators and award information. BICCN Modalities: Defines the modalities profiled by the BICCN. BICCN Techniques: Detailed definition of the techniques used by BICCN investigators. BICCN Species: Species profiled including binomial name and NCBI taxon ID.
(XLSX)

S2 Table Description of data organization by data levels, definitions, and classification. Definition of data levels defined by the BICCN. Columns are detailed definition for each specific modality profiled.
(XLSX)

S3 Table Inventory of BICCN datasets and description of their data level and provenance. List of all datasets produced by BICCN investigators and laboratories. Columns define grant, PI, modality, level of data, description of data, collection name, provenance of data and location in archives, tools used in processing data.
(XLSX)

S4 Table Inventory of BICCN applications and resources, definitions, access identification by RRID and URL. Complete set of software and tools resources generated by the BICCN, including type, RRID, name of resource, location for access and description.
(XLSX)

S1 Text A guide to the BRAIN Initiative Cell Census data ecosystem. (DOCX)

## Abbreviations

BAP: Brain Architecture Project
BCDC: BRAIN Cell Data Center
BDSO: Brain Data Standards Ontology
BICAN: BRAIN Initiative Cell Atlas Network
BICCN: BRAIN Initiative Cell Census Network
BIDS: Brain Imaging Data Structure
BIL: Brain Imaging Library
BossDB: Brain Observatory Storage Service and Database
BRAIN: Brain Research Through Advancing Innovative Neurotechnologies
CAP: complete, accurate, and permanent
CCF: common coordinate framework
CL: Cell Ontology
CT: corticothalamic projecting
CTKE: Cell Type Knowledge Explorer
DANDI: Distributed Archives for Neurophysiology Data Integration
DSP: Data Sciences Platform
DWI: diffusion-weighted imaging
FAIR: Findable, Accessible, Interoperable, and Reusable
GDM: Generative Diffeomorphic Mapping
HCA: Human Cell Atlas
HPC: high-performance computing
IT: intratelencephalic
MCP: Mouse Connectome Project
MRI: magnetic resonance imaging
NeMO: Neuroscience Multi-Omic Data Archive
NHP: nonhuman primate
NWB: Neurodata without Borders
PT: pyramidal tract
RRID: Research Resource Identifier
sc/snRNA-seq: single-cell and single-nucleus RNA-seq
VM: virtual machine
WARP: WDL Analysis Research Pipelines
WDL: Workflow Description Language
