# Supplementary material for: A guide to the BRAIN Initiative Cell Census Network data ecosystem
Source: PLoS Biol. 2023 Jun 30;21(6):e3002133. doi: 10.1371/journal.pbio.3002133 (PMC10313015; doi:10.1371/journal.pbio.3002133)
Supplement: S1 Text — (DOCX) [file pbio.3002133.s005.docx]

**A Guide to the BRAIN Initiative Cell Census Data Ecosystem**

***Supplementary Information***

**BICCN Scientific Outcomes**

Scientific outcomes of the BICCN have resulted in a dramatically increased understanding of the diversity and consistency of cell types within and between species. A major publication summarizing the initial findings of the consortium for a cross-species analysis of primary motor cortex was published in *Nature,* October 6, 2021, (1). That publication contains 11 publications associated with this consortium that have demonstrated:

- A unified molecular genetic catalog of cortical cell types that integrates transcriptome, open chromatin, and DNA methylation maps (2).
- A cross-species analysis that provides a unified taxonomy of transcriptomic types and their hierarchical organization that are conserved from mouse to marmoset and human (1,3).
- Compelling evidence for the epigenomic, transcriptomic, and gene regulatory basis of neuronal phenotypes such as their physiological and anatomical properties, demonstrating the molecular underpinning of neuron types and subtypes (4,5).
- Identification of spatially resolved cell types through *in situ* single-cell transcriptomics (6).
- Development of imaging pipelines and tools for morphometry at whole brain scale, cross modality brain mapping, and resources of single cell morphological cell types (7).
- An extensive genetic toolset for targeting and fate mapping glutamatergic projection neuron types aimed at linking their developmental trajectory to their circuit function (8).
- Brain cell type ontologies and nomenclature systems for describing and manipulating cell types (9,10).

Together, the results establish a unified and mechanistic framework of neuronal cell type organization that integrates multimodal molecular, genetic and spatial information with multi-faceted phenotypic properties.

**BICCN Data Levels**

A primary value of defining data levels with well-defined structure is in the identification of matching appropriate data with use-cases. The general definition for each level is described below. BICCN workgroups evaluated each of 26 modalities to arrive at a more specific level definition as given in Suppl. Table 2.

**
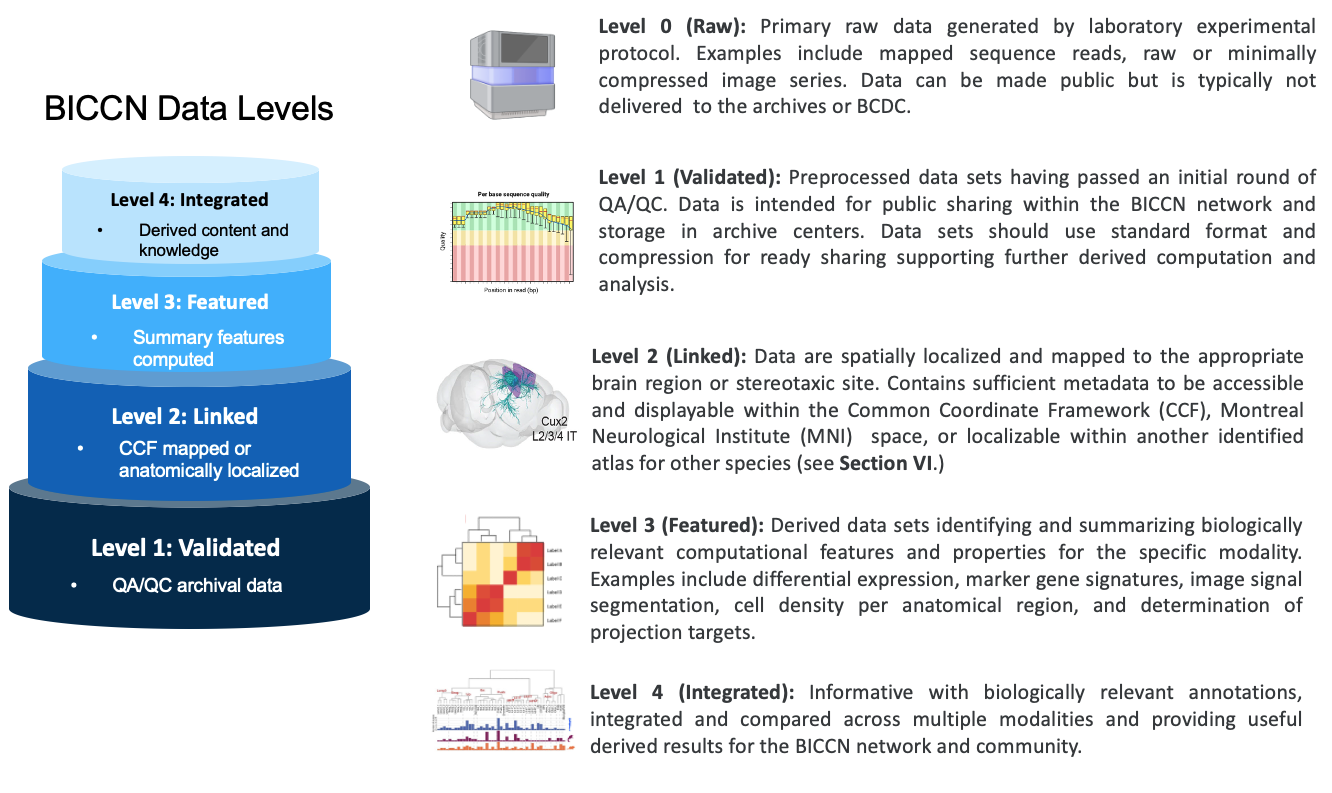
**

**Fig A: Data Levels.** BICCN data are classified by increasing levels of structure and information content. Level 0 (not shown) raw data, Level 1 QC/QA validated, Level 2 CCF-linked or CCF-mapped, Level 3 feature-derived, Level 4 integrated, annotated. Representative use cases are given below.

- **Level 1 (Validated) Use-Case:** A computational analyst wishes to explore differences in alternative splicing and their significance in variability of cell types, comparing between species and in relationship to the chromatin landscape. Requirements: Rigorous QC metrics from transcriptomics and epigenomics modalities. Confidence in mapping alignment is essential (Level 1).

- **Level 2 (Linked)** **Use-Case:** An application developer is building a tool to display long range neuronal connections and cell type specific density distribution in the mouse cortex and corresponding metadata for connection with other modalities. Requirements: Reconstructed and QC neurons registered in the CCF, access to mapping transforms for graphical manipulation and display (Level 2).

-

- **Level 3 (Featured) Use-Case:** User is working with a Human Lung Cell Atlas (11) and wishes to understand differences in cell types found in certain brain regions versus the lung. Requirements: This user requires quantified data sets with relevant genomic features identified. Primary interest will be omics-related data sets with spatial regions identified (Level 3).

-

- **Level 4 (Integrated) Use-Case**: A software developer in an electron microscopy lab is trying to build an application connecting local neuronal morphology with ultrastructural characteristics. Requirements: This user needs spatially resolved local morphology reconstructions with feature quantification in light and EM modalities. (Levels 3 and 4)

**Common Coordinate Frameworks**

**Mouse CCF Enhancements**

Even for the mouse, one of the most widely used model organisms, the best available histological atlases (e.g., the Allen Reference Atlas (12,13) have significant problems and lack *in vivo* MRI or CT data. Thus, it is not possible to directly determine the coordinate system relevant for stereotactic surgery. Usage of auto fluorescent contrast in place of histochemical contrast has resulted in the loss of cytoarchitectonic information, giving rise to controversies about the location of compartmental boundaries. Further, brainstem annotations and volume are truncated. To address these limitations, we have acquired a multimodal data set, including CT scans, MRI (*in vivo*, *ex vivo*), and histology (Nissl, myelin) data in the same animal. We collect histological series using 3 sectioning planes (coronal, axial, sagittal) at 10-µm section thickness. The sectioning preserves the skull to keep brainstem structures intact. The best existing atlases use one sectioning plane (coronal) and 100-µm spacing. We cross-modally register all datasets and use skull landmarks from *in vivo* MRI and CT to define the stereotactic coordinate system. All datasets are available for viewing and download through our in-house web platform.

**Marmoset CCF**

The new marmoset CCF comprises the averaged *in vivo* T2w MRI volumes of 43 marmosets, (36 female and 7 male), *ex vivo* T2w MRI volumes of 45 marmosets (36 female and 5 male), and high-quality Nissl and myelin histological series from two (1 female and 1 male) subjects. Female and male *in vivo* MRI volumes were also averaged separately, creating CCFs for each sex. The segmentation applied to this CCF interpolates between the RIKEN16 and NIH36 marmoset brain atlases, to fill in the gaps in assigning regional identities to all gray and white matter structures. We use the Paxinos37 print atlas as a supplement to fill in the remaining gaps in segmentation with macroscopic level annotations and resolve some mismatches across the atlases. The combined NIH36 and RIKEN16 atlas volume with the refined segmentation applied is then registered to the new marmoset CCF.

**Human CCF**

The Brain Architecture Project (<http://brainarchitecture.org>) is constructing a new CCF for human brains at a previously unprecedented spatial resolution and combining radiological and histological imaging modalities. The new CCF consists of multimodal MRI scans and a series of 3 histological stains (Nissl, myelin, and H&E) carried out on a contiguous set of 20-µm serial sections using the tape-transfer method and reassembled into a 3D multimodal volume. The new CCF rectifies important gaps in previous atlases: The AIBS human reference atlas38 has only 107 histological images. The BigBrain atlas39 from has a complete serial set of approximately 8000 images of 20-µm sections, but only Nissl stained and imaged at low resolution. The new CCF construction pipeline is currently being piloted and refined by two test cases: the creation of a human amygdala and a human hippocampus reference volume.

**Common Coordinate Framework Mapping**

Registration techniques and strategy to a CCF are highly dependent on the specific data modality used. Image centric data, where volumetric regions of the brain have been imaged, allows for spatial registration to CCF using more automated image processing tools (see below). These approaches were further developed by the BICCN and use feature detection, image transformation and resampling, and deformable registration methods (14,15) to obtain quality alignment of source data to the CCFv3.


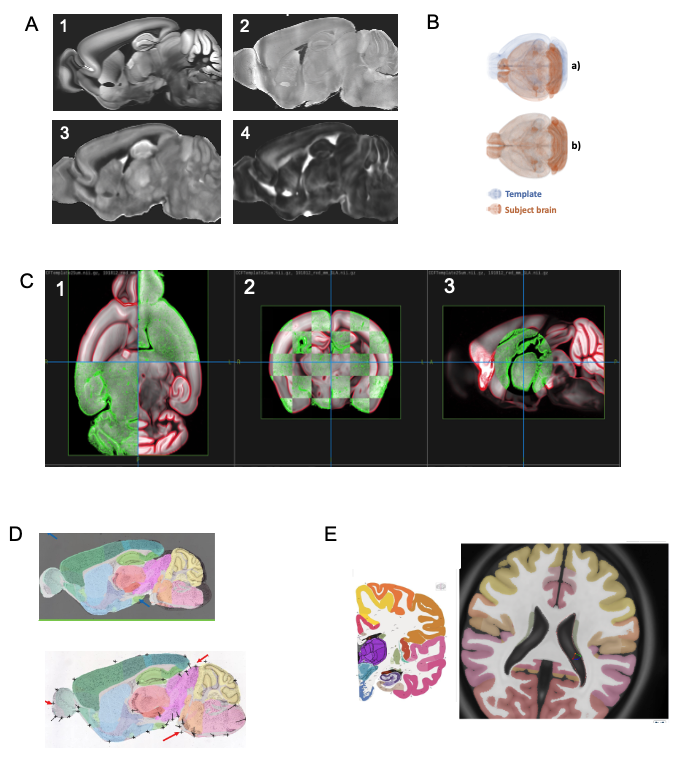


**Fig B: Spatial Registration to Common Coordinate Frameworks.** A) To support image registration to the Allen CCFv3 A1) from different modalities, high resolution templates from MRI and light sheet fluorescence microscopy (LSFM) imaging that are synchronized with CCFv3 have been created (<http://dx.doi.org/10.17632/2svx788ddf.1>). A2) LSFM based template, A3) MRI and A4) FA contrast-based templates. B) Coherent landmark mapping-based registration pipeline mBrainAligner for high-precision cross-modal critical points matching and deformable registration. The tool is available through the web server mBrainAligner-Web. Left panel: The interface of mBrainAligner-Web (http://mbrainaligner.ahu.edu.cn). Right panel: Examples of the subject brain overlaid onto atlas template before a) and after b) registration. C) Software pipeline templates using ANTs (16) (17) for deformable registering data from various imaging modalities (e.g., LSFM, MERFISH, fMOST, MRI) were developed and are available via BCDC, as are custom tools, e.g. Entropy, for interactive evaluation and refinement of alignment results, illustrated here in a use case where fMOST data is mapped to CCFv3, C1) horizontal, C2) coronal, C3) sagittal planes. D) EBRAINS tools for registration of 2D images to the CCFv3. Image shown is from the Allen Mouse Brain Atlas, Experiment 75457579. Upper panel: QuickNII (18) is used to register the section to CCFv3 using affine transformations ([RRID:SCR_016854](https://scicrunch.org/scicrunch/Resources/record/nlx_144509-1/SCR_016854/resolver?q=QuickNII&l=QuickNII)) Lower panel: the registration obtained with QuickNII is refined with VisuAlign ([RRID:SCR_017978](https://scicrunch.org/scicrunch/Resources/record/nlx_144509-1/SCR_017978/resolver?q=VisuAlign&l=VisuAlign)). The atlas is non-linearly deformed over selected target points (black crosses, lower). E) Mapping of human tissue samples is efficiently done using the Cell Locator (<https://github.com/BICCN/cell-locator>), developed in collaboration with Kitware ([www.kitware.com](http://www.kitware.com)). Based on Slicer technology ([www.slicer.org](http://www.slicer.org)), the human reference annotated atlas serves as data mapping and spatially resolved guides for cell type profiling.

**CCF_Streamlines Package**

A Python package ccf_streamlines ([RRID:SCR_022910](https://github.com/alleninstitute/ccf_streamlines)) was developed to map and visualize the isocortical streamlines of the Allen Mouse Common Coordinate Framework (CCF). These streamlines can be used to visualize three-dimensional CCF-aligned data from the mouse isocortex in flattened or two-dimensional representations, as well as to align data in a consistent pia/white matter orientation. The package allows projecting 3D CCF-aligned data to two-dimensional views and to a 3D slab. The package documentation can be found at [https://ccf-streamlines.readthedocs.io/en/latest/index.html](https://nam12.safelinks.protection.outlook.com/?url=https%3A%2F%2Fccf-streamlines.readthedocs.io%2Fen%2Flatest%2Findex.html&data=05%7C01%7C%7C3919e6961a9e458896ac08dab2f88ded%7C32669cd6737f4b398bddd6951120d3fc%7C0%7C0%7C638019077851323138%7CUnknown%7CTWFpbGZsb3d8eyJWIjoiMC4wLjAwMDAiLCJQIjoiV2luMzIiLCJBTiI6Ik1haWwiLCJXVCI6Mn0%3D%7C3000%7C%7C%7C&sdata=AM1H0XK%2B9KzdVWzkD0XSiwg15sqAfwZ9mVHudQMqNn8%3D&reserved=0).

**Generative Diffeomorphic Mapping for image registration and atlas mapping from the Brain Architecture Portal**. For registration and atlas mapping of multimodal imaging datasets (e.g., datasets combining *in* and *ex vivo* imaging and histology in the same animal/subject), the GDM (Generative Diffeomorphic Mapping) registration algorithm can be employed. Briefly, tissue processing procedures such as extraction and fixation cause brain tissue deformation (19) (20) and unguided reconstruction of serial sections leads to accumulated long-range distortions (21). Diffeomorphic mapping emerged to overcome these challenges (22). Our approach to atlas mapping and registration is a generative probabilistic model, where a synthetic stack of 2D microscopy images is formed as a sequence of transforms of a 3D image, plus a noise model describing variability. Transforms include diffeomorphic spatial warping and contrast changes. Mapping to common coordinates is maximum a posteriori (23,24) estimation, and enables reconstruction of 3D and 2D datasets. We quantify tissue distortion with the derivative of spatial maps (morphometry) (25) and account for scale changes when quantifying cell density or number (23). We support *in vivo* and *ex vivo* MRI, atlas annotations including independently derived segmentation (24) and multiple stained sections. This framework enables us to jointly analyze multiple MRI contrasts and histology data, providing ground truth data for evaluation of MR models. MRI-constrained reconstruction developed specifically for the marmoset data in our pipeline shows improved accuracy over the baseline method and reduced deformable metric cost. The local scale change between postmortem MRI volumes and reassembled 3D histological volumes from the tape-transfer method is small (~2-3% medial absolute scale change), less than the pre-mortem to postmortem change (~6-10%, measured using the Jacobian determinant of metric tensor relating the corresponding spaces (26). We are (7)currently building a registration toolbox page into the Brain Architecture portal, which will allow users to register their image series to any of the CCFs available on the Brain Architecture portal using an online interface.

**BICCN Image Processing Pipelines**

The Image and Multi-Morphology Pipeline (4) (7) accesses raw images from the BIL archive (Section IV.2) and implements the full pipeline of conversion, processing, morphometry generation, registration and mapping, release, and analysis. The pipeline is hosted on an open cloud platform that features collaborative processing and synergetic computing among various clients, and web interfaces. All data on the server can be accessed through MorphoHub (27), a petabyte-scale multi-morphometry management system and integrates the three largest whole-brain full morphology datasets (7), and SEU-Allen Institute of neuron reconstruction of the whole brain (28,29). The pipeline starts with downloading whole-brain images from public repositories (BrainImageLibrary), then conversion, processing, morphometry generation, registration and mapping, release, and analysis. The pipeline is hosted on an open cloud platform that features collaborative processing and synergetic computing among various clients, including Vaa3D, mobile application Hi5, VR headset system TeraVR, high-performance clusters, and web interfaces, resulting in a dynamic, fast while accurate image and morphometry processing pipeline. All data on the server can be accessed through MorphoHub (27), a Petabyte-scale multi-morphometry management system. As an open-source pan-neuron table of morphology and connectivity, NeuroXiv integrates the three largest whole-brain full morphology datasets.

**Brain Observatory Storage Service and Database**

Brain Observatory Storage Service and Database (BossDB, [RRID:SCR_017273](https://scicrunch.org/resources/data/record/nlx_144509-1/SCR_017273/resolver?q=SCR_017273&l=SCR_017273&i=rrid:scr_017273), [https://bossdb.org](https://bossdb.org/)) is a volumetric, cloud-based data ecosystem for 3D and 4D neuroimaging data (30). As the most recent BICCN archive, BossDB focuses primarily on storing volumetric electron microscopy (EM) and X-ray microtomography (XRM) datasets generated as a part of the BRAIN Initiative. BossDB stores high- resolution, multi-channel image data with registered segmentations, annotations, and meshes, and connects to several community resources for data access and data visualization. BossDB also stores connectomics datasets and contains several software tools and interfaces for querying and searching connectomes (31) . The BossDB ecosystem allows for storing, accessing, and processing multidimensional and volumetric neuroscience datasets through scalable cloud-based resources, and makes use of Amazon Web Services capabilities that ensure available and scalable endpoints, data caching and load balancing, and durable multi-tier data storage. A well-documented interface and API supports a suite of tools, including a Python based software development kit (SDK) that allows a user to easily ingest, validate, visualize, and query neuroscience data from any data generator, making it possible for scientists to discover and share insights on these massive datasets (31). For further information see Suppl. Materials and [BossDB.org | Get Started](https://bossdb.org/get-started).

BossDB provides several data ingest paradigms and supports the ingest of several image data formats, where the image and segmentation data is stored in cuboids within a cloud-based object data store. Following data ingestion into BossDB, multiple down-sampled image volume copies are generated and stored to ensure performant data access at multiple image resolutions. BossDB uses a hierarchical organizational structure where it organizes datasets by "collection", "experiment", and "channel". Collections are the top structure containing metadata like the laboratory name and date of creation. Experiments are mid-level structures containing more metadata about the imaging modality, image extent and coordinate frame for the imaged tissue samples (where data exists), resolution levels, and imaging voxel size. Channels, the lowest level structures, contain the volumetric data.

Datasets on the BossDB website (https://[bossdb.org/projects](https://bossdb.org/projects)) are provided as projects associated with major publications, are publicly available and free to download, and all tools listed are open-source and available for public use (<https://bossdb.org/tools>). The BossDB provides extensive documentation on the infrastructure, the interfaces, and the various tools that can be used to ingest, access, and visualize volumetric data. A BossDB Cookbook repository (<https://github.com/aplbrain/bossdb_cookbook>) is a collection of introductory notebooks and examples for interfacing with the BossDB system. For further information see <https://bossdb.org/get-started>.

**Brain Architecture Portal**

All datasets containing high-resolution 2D images from the Mitra Lab (<http://brainarchitecture.org/mitra-lab>) , BICCN collaborators (32), and colleagues from other projects (33–36) are available for display on the Brain Architecture web portal. We have extensive experience in serving peta-voxels of light microscopic data on the web. The Brain Architecture web portal has been in continuous operation since 2012 and receives 500-1000 unique visitors/month. Datasets are served into species and experiment-type specific pages, accessible from the front landing page. For example, there is the capability to filter mouse cell distribution datasets via free text search of metadata for keywords and mouse projection and connectivity datasets via injection region or tracer. The viewer has in-built capability to display overlays of regional compartments, points indicating cell bodies post cell detection, and skeletons and shaded pixels indicating neuritis post DMM++4 process detection and skeletonization on 2D sections of atlas mapped brains. To date, the viewer displays >2 peta-voxels of images using an Angular framework with MySQL DB queries for image file loading directed by Django-based APIs. The viewer can display data at multiple resolutions, with zoom to super resolution capability, beyond the native in-plane 0.46-µm resolution of the images.

In addition to thousands of viewable datasets across multiple projects, all software tool sets employed in projects involving the Mitra laboratory image analytics pipeline, including GDM registration and atlas mapping cell detection3, and process detection and skeletonization via DMM++4, will be available both in interactive versions on the Brain Architecture web portal, and for download (of both source code and documentation) on Github and Bitbucket repositories; buttons to access these can be found on the new front page. Interactive analytic tool sets will have their own dedicated pages on the Brain Architecture web portal and will be open to use for any user who creates a free account. All downloads from Github and Bitbucket repositories will be unrestricted, in keeping with Open-Source code practices.

**BICCN Tools and Resources**

Many essential tools and resources were developed throughout the BICCN and are summarized below and on the portal https://biccn.org/tools.

#### **Epiviz** ([RRID:SCR_022796](https://www.cbcb.umd.edu/software/epiviz)**),** an interactive visualization tool for functional genomics data; *Brainome* (<https://brainome.ucsd.edu/annoj/BICCN_MOp/>) a genome browser to visualize the cell type-specific transcriptomes and epigenomes of cell types from the mouse MOp.

#### **Catlas** ([RRID:SCR_018690](http://catlas.org/mousebrain/)), which provides maps of accessible chromatin in >800,000 individual cells from 45 regions spanning the adult mouse isocortex, olfactory bulb, hippocampus, and cerebral nuclei.

#### **Chan Zuckerberg Initiative** (CZI, [RRID:SCR_021059](https://scicrunch.org/resources/data/record/nlx_144509-1/SCR_021059/resolver?q=SCR_021059&l=SCR_021059&i=rrid:scr_021059)) [CZ CELL x GENE](https://cellxgene.cziscience.com) is a web-based interface for exploring high dimensional datasets along categorical, continuous and spatial dimensions, as well as feature annotation and hosts several of the molecular datasets of the BICCN, Human Cell Atlas (<https://www.humancellatlas.org>, HCA, [RRID:SCR_016530](https://scicrunch.org/scicrunch/Resources/record/nlx_144509-1/SCR_016530/resolver?q=human%20cell%20atlas&l=human%20cell%20atlas)), and and other consortia including an atlas of cortical arealization in developing human [CZ CELL x GENE](https://cellxgene.cziscience.com/collections/c8565c6a-01a1-435b-a549-f11b452a83a8) (37).

#### **Cytosplore Viewer** ([RRID:SCR_018330](https://scicrunch.org/resources/data/record/nlx_144509-1/SCR_018330/resolver?q=SCR_018330&l=SCR_018330&i=rrid:scr_018330) is a stand-alone application (Windows and MacOS) for interactive visual exploration of multi-species and cross-omics single cell data in several BICCN data resources. It supports interactive exploration of cellular hierarchies, gene expression and metadata of individual cells, and it allows computation of differential statistics between cell selections or clusters, and across species. Cytosplore Viewer enables linked cross-omics, cross-species comparison between matched gene expression, chromatin state and DNA methylation data through linkout to the UCSC genome browser.

#### **CloudVolume** ([RRID:SCR_022820](https://github.com/seung-lab/cloud-volume)) is a Python package that allows for easy reading and writing of image data in the neuroglancer precomputed format,

#### (<https://github.com/google/neuroglancer/tree/master/src/neuroglancer/datasource/precomputed>). The precomputed format allows for efficient visualization of large 3D images by exploiting the fact that at any given moment, users typically only need to view a small subset of an image, i.e. a certain spatial area at a certain resolution. Many of the datasets hosted at BossDB (<https://bossdb.org/projects>) are available in the precomputed format, and therefore the raw digital data can be read in Python using CloudVolume.

#### **MetaNeighbor** (38,39) ([RRID:SCR_016727](https://scicrunch.org/resources/data/record/nlx_144509-1/SCR_016727/resolver?q=SCR_016727&l=SCR_016727&i=rrid:scr_016727))is a method for assessing the replicability of single cell data, used in a number of key BICCN publications (e.g., (2,3) to validate cell types and perform quality control. Rather than merging data, MetaNeighbor holds data independent and tests the degree to which cell-types in one dataset can be used to characterize the cell-types in another dataset. This highly scalable procedure can be easily applied to test the specific replicability of genes sets. Because the method does not modify the underlying data, it is useful for comparisons where strong real potential differences exist (e.g., cross-species assessment). The R version of MetaNeighbor is available through Bioconductor at

#### <https://www.bioconductor.org/packages/release/bioc/html/MetaNeighbor.html.>

#### The development versions are available on GitHub at <https://github.com/gillislab/MetaNeighbor> (R version) and <https://github.com/gillislab/pyMN> (Python version). The related method MetaMarkers, provides meta-analytic marker sets across the BICCN data corpus (39).

#### **NS-Forest** ([RRID:SCR_018348](https://github.com/JCVenterInstitute/NSForest)) is a Python package that identifies minimum sets of marker genes for cell types identified in sc/snRNA-seq datasets (40,41). The method is based on the selection of informative gene expression features using random forest machine learning and includes a binary scoring step to enrich for genes that show binary on-off expression patterns, which are especially useful for downstream applications like spatial transcriptomics. NS-Forest markers have been used to annotate cell types identified in BICCN datasets (3) and form the basis of cell type definition in the Brain Data Standards Ontology described below and for visualization in the CTKE.

#### **FR-Match** (<https://github.com/JCVenterInstitute/FRmatch>) is an R package for matching cell types across datasets based on a novel application of the Friedman-Rafsky (FR) test, a non-parametric statistical test for multivariate data comparison, in the context of single cell clustering results (42). FR-Match has been validated for matching single nucleus and single cell datasets, matching data from SmartSeq and 10X Genomics platforms, and matching single cell and spatial transcriptomics datasets using BICCN datasets (43), illustrating the value of the BICCN data ecosystem for novel methods development.

#### **Single Cell Portal (**[RRID:SCR_014816](https://singlecell.broadinstitute.org/single_cell)) is a portal to visualize, share, and disseminate single-cell data. As a self-service application, scientists upload data programmatically or use a wizard-driven UI with the help of extensive support (help desk, documentation wiki, and API swagger). Studies allow for a collection of rich interactive visualizations and customizations and are findable through natural language search or curated search facets. Single Cell Portal includes an end-to-end set of functionalities including private, sharing, reviewer, and public settings. This portal is generally available to the scientific community and houses collections associated with the BICCN, the Human Cell Atlas project, the Alexandria project, and COVID-19 research.

**BICCN FAIR Data Practices**

The Findability of pipelines is enabled using RRIDs to give pipelines unique, explicit identifiers. These pipelines are under continuous development and so semantic versioning is used to refer to versions and specify a specific commit of the code. The BICCN Portal hosts a page that collects links to all resources (code repositories, Terra workspaces, publications, and documentation) in one place. To assure Accessibility, the BCDC hosts pipelines in multiple community resources including public GitHub repositories (for software engineers), Dockstore (for computational biologists), and Terra (installed and ready to run for those without local infrastructure or who want to use scalable cloud resources). Pipelines are open-access and freely licensed to encourage not-for-profit as well as for-profit use. All pipelines are fully documented and are preinstalled into Terra with test data and expected outputs to remove the requirement of complicated installations or infrastructure requirements. Interoperability for pipelines is addressed for both infrastructures to run pipelines as well as interoperability with other tools or pipelines. Execution interoperability is encouraged by using a modern workflow language (WDL) that separates the code performing scientific tasks from code for orchestrating the pipeline on infrastructure (contained in workflow execution programs). WDL can be executed by multiple workflow execution programs including [Cromwell](https://cromwell.readthedocs.io/en/develop/), a portable execution engine leveraged in high-throughput sequencing settings that can be launched in many environments. To encourage interoperability with other tools and pipelines, pipelines leverage *de facto* and [GA4GH](https://www.ga4gh.org/) ([www.ga4gh.org](http://www.ga4gh.org/)) standards and preference leveraging community developed tools over creating new tools. Finally, all these activities come together to enable essential Reproducible science.

**Brain Data Standards Ontology**

The BDSO approach is described in detail in (44) not only makes it scalable, but also lowers human error (as compared to manually creating the ontology). These features are crucial as we scale up to whole brains in the new phase described in the next section. BDSO is developed based on the OBO Foundry (45) and FAIR (46) principles; as such, the BDSO is fully compliant with OBO Foundry standards and has been included as an OBO Foundry ontology. The methods we described for creating BDSO were made to be relatively generalizable, allowing future projects to adapt them with BDSO serving as an example.

In their native formats, ontologies are not practical for driving web applications. The BDSO loads into an instance of the graph database Neo4j using a standard pipeline for logical projection of OWL into a Neo4j property graph <https://doi.org/10.5281/zenodo.7082530>, using standard ontology queries to add tags to drive faceted searching for neurons by type (GABAergic), morphology (Chandelier) location (Primary Motor Cortex) and species. The structure of the knowledge graph is used to populate and index documents in a SOLR document store, enabling enhanced full-text search and content retrieval for the CTKE.

The BDSO's code base is available at GitHub ([RRID:SCR_022822](https://github.com/obophenotype/brain_data_standards_ontologies)) including documentation of the full technology stack and details of the approach. The latest release of the ontology is available for download from <http://purl.obolibrary.org/obo/pcl/bds/bds.owl> and is hosted on the EMBL-EBI ontology lookup service OLS ([http://ceur-ws.org/Vol-1546/paper_29.pdf](http://purl.obolibrary.org/obo/pcl/bds/bds.owl)) at <https://www.ebi.ac.uk/ols/ontologies/pcl>. OLS provides ontology search, browsing, visualization capabilities and enables web services driven programmatic access to the BDSO.

**References**

1. BRAIN Initiative Cell Census Network (BICCN). A multimodal cell census and atlas of the mammalian primary motor cortex. Nature. 2021 Oct;598(7879):86–102.

2. Yao Z, Liu H, Xie F, Fischer S, Adkins RS, Aldridge AI, et al. A transcriptomic and epigenomic cell atlas of the mouse primary motor cortex. Nature. 2021 Oct;598(7879):103–10.

3. Bakken TE, Jorstad NL, Hu Q, Lake BB, Tian W, Kalmbach BE, et al. Comparative cellular analysis of motor cortex in human, marmoset and mouse. Nature. 2021 Oct;598(7879):111–9.

4. Li YE, Preissl S, Hou X, Zhang Z, Zhang K, Qiu Y, et al. An atlas of gene regulatory elements in adult mouse cerebrum. Nature. 2021 Oct;598(7879):129–36.

5. Muñoz-Castañeda R, Zingg B, Matho KS, Chen X, Wang Q, Foster NN, et al. Cellular anatomy of the mouse primary motor cortex. Nature. 2021 Oct;598(7879):159–66.

6. Zhang M, Eichhorn SW, Zingg B, Yao Z, Cotter K, Zeng H, et al. Spatially resolved cell atlas of the mouse primary motor cortex by MERFISH. Nature. 2021 Oct;598(7879):137–43.

7. Peng H, Xie P, Liu L, Kuang X, Wang Y, Qu L, et al. Morphological diversity of single neurons in molecularly defined cell types. Nature. 2021 Oct;598(7879):174–81.

8. Matho KS, Huilgol D, Galbavy W, He M, Kim G, An X, et al. Genetic dissection of the glutamatergic neuron system in cerebral cortex. Nature. 2021 Oct 7;598(7879):182–7.

9. Gillespie TH, Tripathy SJ, Sy MF, Martone ME, Hill SL. The Neuron Phenotype Ontology: A FAIR approach to proposing and classifying neuronal types. Neuroinformatics. 2022 Jul;20(3):793–809.

10. Miller JA, Gouwens NW, Tasic B, Collman F, van Velthoven CT, Bakken TE, et al. Common cell type nomenclature for the mammalian brain. Elife [Internet]. 2020 Dec 29;9. Available from: http://dx.doi.org/10.7554/eLife.59928

11. Travaglini KJ, Nabhan AN, Penland L, Sinha R, Gillich A, Sit RV, et al. A molecular cell atlas of the human lung from single-cell RNA sequencing. Nature. 2020 Nov;587(7835):619–25.

12. Wang Q, Ding S-L, Li Y, Royall J, Feng D, Lesnar P, et al. The Allen mouse brain common coordinate framework: A 3D reference atlas. Cell. 2020 May;181(4):936-953.e20.

13. Oh SW, Harris JA, Ng L, Winslow B, Cain N, Mihalas S, et al. A mesoscale connectome of the mouse brain. Nature. 2014 Apr;508(7495):207–14.

14. Goshtasby AA. Image Registration Methods. In: Image Registration. London: Springer London; 2012. p. 415–34. (Advances in computer vision and pattern recognition).

15. Zitová B, Flusser J. Image registration methods: a survey. Image Vis Comput. 2003 Oct;21(11):977–1000.

16. Avants BB, Epstein CL, Grossman M, Gee JC. Symmetric diffeomorphic image registration with cross-correlation: evaluating automated labeling of elderly and neurodegenerative brain. Med Image Anal. 2008 Feb;12(1):26–41.

17. Tustison NJ, Cook PA, Holbrook AJ, Johnson HJ, Muschelli J, Devenyi GA, et al. The ANTsX ecosystem for quantitative biological and medical imaging. Sci Rep. 2021 Apr 27;11(1):9068.

18. Puchades MA, Csucs G, Ledergerber D, Leergaard TB, Bjaalie JG. Spatial registration of serial microscopic brain images to three-dimensional reference atlases with the QuickNII tool. PLoS One. 2019 May 29;14(5):e0216796.

19. Mouritzen Dam A. Shrinkage of the brain during histological procedures with fixation in formaldehyde solutions of different concentrations. J Hirnforsch. 1979;20(2):115–9.

20. Schulz G, Crooijmans HJA, Germann M, Scheffler K, Müller-Gerbl M, Müller B. Three-dimensional strain fields in human brain resulting from formalin fixation. J Neurosci Methods. 2011 Oct 30;202(1):17–27.

21. Malandain G, Bardinet E, Nelissen K, Vanduffel W. Fusion of autoradiographs with an MR volume using 2-D and 3-D linear transformations. Neuroimage. 2004 Sep;23(1):111–27.

22. Bajcsy R, Lieberson R, Reivich M. A computerized system for the elastic matching of deformed radiographic images to idealized atlas images. J Comput Assist Tomogr. 1983 Aug;7(4):618–25.

23. Schmitz C, Hof PR. Design-based stereology in brain aging research. In: Brain Aging. CRC Press; 2007. p. 63–96.

24. Woodward A, Hashikawa T, Maeda M, Kaneko T, Hikishima K, Iriki A, et al. The Brain/MINDS 3D digital marmoset brain atlas [Internet]. bioRxiv. bioRxiv; 2017. Available from: http://dx.doi.org/10.1101/228676

25. Ashburner J, Friston KJ. Voxel Based Morphometry. In: Encyclopedia of Neuroscience. Elsevier; 2009. p. 471–7.

26. Lee BC, Lin MK, Fu Y, Hata J, Miller MI, Mitra PP. Multimodal cross-registration and quantification of metric distortions in marmoset whole brain histology using diffeomorphic mappings. J Comp Neurol. 2021 Feb;529(2):281–95.

27. Jiang S, Wang Y, Liu L, Ding L, Ruan Z, Dong H-W, et al. Petabyte-scale multi-morphometry of single neurons for whole brains. Neuroinformatics. 2022 Apr;20(2):525–36.

28. Winnubst J, Bas E, Ferreira TA, Wu Z, Economo MN, Edson P, et al. Reconstruction of 1,000 projection neurons reveals new cell types and organization of long-range connectivity in the mouse brain [Internet]. bioRxiv. bioRxiv; 2019. Available from: http://dx.doi.org/10.1101/537233

29. Gao L, Liu S, Gou L, Hu Y, Liu Y, Deng L, et al. Single-neuron projectome of mouse prefrontal cortex. Nat Neurosci. 2022 Apr;25(4):515–29.

30. Hider R Jr, Kleissas D, Gion T, Xenes D, Matelsky J, Pryor D, et al. The Brain Observatory Storage Service and Database (BossDB): A cloud-native approach for petascale neuroscience discovery. Front Neuroinform. 2022 Feb 15;16:828787.

31. Matelsky JK, Rodriguez LM, Xenes D, Gion T, Hider R, Wester BA, et al. An integrated toolkit for extensible and reproducible neuroscience. Annu Int Conf IEEE Eng Med Biol Soc. 2021 Nov;2021:2413–8.

32. Jeong M, Kim Y, Kim J, Ferrante DD, Mitra PP, Osten P, et al. Comparative three-dimensional connectome map of motor cortical projections in the mouse brain. Sci Rep. 2016 Feb 2;6(1):20072.

33. Lin MK, Takahashi YS, Huo B-X, Hanada M, Nagashima J, Hata J, et al. A high-throughput neurohistological pipeline for brain-wide mesoscale connectivity mapping of the common marmoset. Elife [Internet]. 2019 Feb 5;8. Available from: http://dx.doi.org/10.7554/eLife.40042

34. Majka P, Bai S, Bakola S, Bednarek S, Chan JM, Jermakow N, et al. Open access resource for cellular-resolution analyses of corticocortical connectivity in the marmoset monkey. Nat Commun. 2020 Feb 28;11(1):1133.

35. Hashikawa T, Iriki A, Okano HJ, Sasaki E, Okano H. Introduction. In: The 3-Dimensional Atlas of the Marmoset Brain. Tokyo: Springer Japan; 2018. p. 1–32.

36. Chen Y, McElvain LE, Tolpygo AS, Ferrante D, Friedman B, Mitra PP, et al. An active texture-based digital atlas enables automated mapping of structures and markers across brains. Nat Methods. 2019 Apr;16(4):341–50.

37. Bhaduri A, Sandoval-Espinosa C, Otero-Garcia M, Oh I, Yin R, Eze UC, et al. An atlas of cortical arealization identifies dynamic molecular signatures. Nature. 2021 Oct;598(7879):200–4.

38. Crow M, Paul A, Ballouz S, Huang ZJ, Gillis J. Characterizing the replicability of cell types defined by single cell RNA-sequencing data using MetaNeighbor. Nat Commun. 2018 Feb 28;9(1):884.

39. Fischer S, Gillis J. How many markers are needed to robustly determine a cell’s type? iScience. 2021 Nov 19;24(11):103292.

40. Aevermann B, Zhang Y, Novotny M, Keshk M, Bakken T, Miller J, et al. A machine learning method for the discovery of minimum marker gene combinations for cell type identification from single-cell RNA sequencing. Genome Res. 2021 Oct;31(10):1767–80.

41. Aevermann BD, Novotny M, Bakken T, Miller JA, Diehl AD, Osumi-Sutherland D, et al. Cell type discovery using single-cell transcriptomics: implications for ontological representation. Hum Mol Genet. 2018 May 1;27(R1):R40–7.

42. Zhang Y, Aevermann BD, Bakken TE, Miller JA, Hodge RD, Lein ES, et al. FR-Match: robust matching of cell type clusters from single cell RNA sequencing data using the Friedman-Rafsky non-parametric test. Brief Bioinform [Internet]. 2021 Jul 20;22(4). Available from: http://dx.doi.org/10.1093/bib/bbaa339

43. Zhang Y, Aevermann B, Gala R, Scheuermann RH. Cell type matching in single-cell RNA-sequencing data using FR-Match. Sci Rep. 2022 Jun 15;12(1):9996.

44. Tan SZK, Kir H, Aevermann BD, Gillespie T, Harris N, Hawrylycz MJ, et al. Brain Data Standards - A method for building data-driven cell-type ontologies. Sci Data. 2023 Jan 24;10(1):50.

45. Jackson RC, Matentzoglu N, Overton JA, Vita R, Balhoff JP, Buttigieg PL, et al. OBO Foundry in 2021: Operationalizing open data principles to evaluate ontologies [Internet]. bioRxiv. bioRxiv; 2021. Available from: http://dx.doi.org/10.1101/2021.06.01.446587

46. Wilkinson MD, Dumontier M, Aalbersberg IJJ, Appleton G, Axton M, Baak A, et al. The FAIR Guiding Principles for scientific data management and stewardship. Sci Data. 2016 Mar 15;3:160018.
